# Heart Rate Variability Integrated into a Longitudinal Multimodal Model to Predict Delayed Cerebral Ischemia After Aneurysmal Subarachnoid Hemorrhage

**DOI:** 10.3390/bioengineering13080857

**Published:** 2026-07-25

**Authors:** Valerie C. Schütz, Jens M. Boss, Corinne Inauen, Stefan Y. Bögli, Tilman Beck, Emanuela Keller, Jan F. Willms

**Affiliations:** 1 Neurocritical Care Unit, Institute of Intensive Care Medicine, Clinical Neuroscience Center, University Hospital Zurich and University of Zurich, 8091 Zurich, Switzerland; valeriecornelia.schuetz@uzh.ch (V.C.S.); jens.michael.boss@gmail.com (J.M.B.); corinne.inauen@usz.ch (C.I.); stefanyu.boegli@usz.ch (S.Y.B.); tilman.beck@usz.ch (T.B.); 2 Department of Neurology, University Hospital Tulln, 3430 Tulln, Austria; 3 Department of Neurology, Clinical Neuroscience Center, University Hospital Zurich and University of Zurich, 8091 Zurich, Switzerland; 4 Neurocritical Care Unit, Department of Neurosurgery and Institute of Intensive Care Medicine, Clinical Neuroscience Center, University Hospital Zurich and University of Zurich, 8091 Zurich, Switzerland; emanuela.keller@usz.ch

**Keywords:** subarachnoid hemorrhage, machine learning, heart rate variability, delayed cerebral ischemia

## Abstract

Background: Delayed cerebral ischemia (DCI) is a major cause of morbidity after aneurysmal subarachnoid hemorrhage (aSAH), yet early prediction remains difficult, especially in comatose patients. Existing risk scores are largely static and do not capture dynamic pathophysiological changes. Heart rate variability (HRV), reflecting autonomic nervous system activity, may provide additional predictive information. We investigated whether integrating HRV into a longitudinal multimodal model improves DCI prediction. Methods: In this prospective cohort study, continuous ECG data were collected from patients with aSAH admitted to a tertiary neurocritical care unit between 2016 and 2024. HRV features were computed in real time from high-resolution ECG recordings and combined with demographic, clinical, laboratory, and blood gas variables. Four prediction models were evaluated: static clinical variables, laboratory/blood gas data, HRV features, and a multimodal combined model. Performance was assessed using cross-validation, anchored ROC analysis, and leave-one-out simulations. Results: Among 101 eligible patients, 35 (34.7%) developed DCI. LFNorm showed the best univariate HRV performance (ROC AUC 0.64). HRV features and laboratory/blood gas models achieved ROC AUCs of 0.60 and 0.63, respectively. The multimodal model achieved the highest overall performance (ROC AUC 0.68), with HRV features accounting for more than half of the model’s feature importance. Time-resolved analyses further demonstrated that the contribution of HRV varied across the clinical course, providing the greatest incremental value during the later phase preceding DCI. Conclusions: Integrating HRV into a multimodal prediction framework provides additional time-dependent information for DCI risk estimation in patients with aSAH.

## 1. Introduction

Worldwide, almost 700,000 patients suffer from aneurysmal subarachnoid hemorrhage (aSAH) annually [1]. After the primary brain injury due to aneurysm rupture, delayed cerebral ischemia (DCI) occurs in up to one-third of the patients and is a major cause of death and disability [2,3]. The prediction and early detection of DCI in aSAH is critical to prevent secondary infarctions and is particularly challenging in comatose patients [4,5]. Various scores are available to predict the outcome and the probability of developing DCI, including the Hunt and Hess, the World Federation of Neurosurgical Societies (WFNS), Fisher, Barrow Neurological Institute (BNI), and VASOGRADE scales [6,7,8,9,10]. However, these scores have limited predictive value. This is primarily due to the fact that they are determined at the time of patient admission and are thus static and do not take into account the dynamically changing factors that contribute to the development of DCI. Recently, a longitudinal machine learning (ML)-based algorithm for DCI risk prediction has been developed. This algorithm is based on predictors such as patient characteristics and 45 laboratory findings and values from arterial blood gas analysis [11]. Two models were trained: a static model using demographic and SAH grading scores at admission and a dynamic model using regularly sampled laboratory and blood gas analysis data. A combined model using a voting approach to predict DCI attained an ROC AUC of 0.73 ± 0.05 [11].

Heart rate variability (HRV) reflects the interplay between sympathetic and parasympathetic activity. It provides a non-invasive and continuous measure of autonomic nervous system balance [12,13]. HRV is a biomarker in sepsis-associated systemic inflammatory response syndrome (SIRS) [14]. SAH following aneurysm rupture has been shown to induce a cerebral and systemic inflammatory response, which is associated with the development of cerebral vasospasm and DCI [15,16]. It can thus be concluded that HRV monitoring might have the potential to predict DCI. A few studies have previously examined the potential of HRV as a biomarker, with initial findings indicating a satisfactory sensitivity, though with limited specificity [17,18,19,20,21]. This suggests the need for approaches that combine HRV with additional multimodal parameters to enhance diagnostic accuracy.

ICU Cockpit^®^ is a secure IT platform currently in use in the Neurocritical Care Unit (NCCU) at the University Hospital Zurich. It collects high-resolution multimodal data, supports computational disease modeling, and enables real-time validation and deployment of decision support systems in critical care. Uniquely, it allows algorithms—such as those for HRV analysis to predict DCI—to be seamlessly integrated into the live data stream, providing real-time clinical decision support and predictive insights at the bedside [22].

This project hypothesizes that incorporating specific features extracted from high-temporal-resolution biosignals, such as HRV, can improve the accuracy of an existing longitudinal model for predicting DCI, which has previously been based only on low-resolution data.

The specific steps of the present project were: (1) to develop an algorithm for the real-time computation of HRV features, (2) to analyze their value for DCI prediction (a) as a single biomarker and (b) in combination with the dynamic input features from the pre-existing model for DCI prediction based on predictors with low temporal resolution that was already running in the ICU Cockpit^®^ live environment, and (3) to implement them into the live data stream.

## 2. Methods

The authors adhere to the TRIPOD (transparent reporting of a multivariable prediction model for individual prognosis or diagnosis) reporting guidelines.

### 2.1. Participants and Data Source

The data were prospectively collected from patients with aSAH who were admitted to the neurosurgical intensive care unit at the University Hospital of Zurich in Switzerland between November 2016 and April 2024. Continuous electrocardiography (ECG) data were collected at 500 Hz using a Philips IntelliVue monitor (MX750, Böblingen, Germany). The data collection was carried out via a CNS data collector (Moberg ICU Solutions, Ambler, PA, USA), and the high-resolution data were processed and stored by the ICU Cockpit^®^, our IT platform [22]. The study received approval from the ethics committee of Kanton Zurich (BASEC Nr. 2025-02422), Switzerland. Patients were included in this study after written consent had been obtained from the patients or their legal representatives.

### 2.2. Diagnostics and Treatment

Clinical management was guided by the American Heart Association guidelines [23]. Diagnostics and treatment are described in detail elsewhere [11]. Importantly, magnetic resonance imaging (MRI) or computed tomography (CT) scans, including CT–angiography/perfusion, were performed if patients met the criteria for delayed neurological deterioration (DND), defined as >2-point change in Glasgow Coma Scale (GCS) or new focal neurological deficit lasting >1 h, not associated with aneurysmal coiling or clipping [3]. In unconscious or sedated patients, multimodal neuromonitoring was performed, incorporating brain tissue oxygen measurements and cerebral microdialysis. In these patients, a decrease in the partial pressure of oxygen in the brain tissue (ptiO_2_) < 20 mmHg and/or an increase in the lactate/pyruvate coefficient above 40 led to one of the above-mentioned imaging techniques [4,11].

### 2.3. Prediction Target, Predictors, Pre-Processing, and Missing Data

The outcome, i.e., the prediction target, remained the same as defined for the pre-existing DCI prediction algorithm based on low-resolution input features [11], and was the first occurrence of DCI. DCI was defined as new infarctions detected on MRI or CT scans, and/or a confirmed perfusion deficit detected in perfusion CT or MRI between day 4 and day 14 after the onset of symptoms (not present on imaging performed within 24 to 48 h after aneurysm occlusion, and not attributable to other causes). The pre-existing data model for DCI prediction comprised a dataset with demographic characteristics (e.g., age and sex), admission neurological status GCS, comorbidities such as diabetes, standard aSAH gradings, including the Hunt and Hess scale, modified Fisher scale, WFNS grading system, and BNI scale, as well as 30 laboratory findings and 15 values from arterial blood gas analysis routinely collected in patients with aSAH. DCI was diagnosed as part of routine clinical care according to the predefined diagnostic criteria described above. HRV-derived features and prediction model outputs were generated retrospectively after completion of patient recruitment and were, therefore, unavailable to treating physicians and outcome assessors. Pre-processing was performed, and missing data were handled as described by Willms et al. [11].

### 2.4. Data Modeling

Data modeling for the pre-existing algorithm based on low-resolution data is described in detail by Willms et al. [11]. In principle, four dynamic DCI classification models were trained, all anchored at the time of DCI: (1) a model based on static variables recorded at ICU admission (STATIC), (2) a model based on results from laboratory and blood gas analysis (LAB/BGA), (3) a model based on additional input features, which were derived from the computation of HRV, and (4) a multimodal model combining all of the aforementioned features (COMBINED). The features of both dynamic variants (LAB/BGA and HRV) were extended with aggregates of different time windows to account for temporal variations and noise in the data. Multiple classifiers, including Logistic Regression, random forest, histogram-based Gradient Boosting Machines, Extreme Gradient Boosting, and Extremely Randomized Trees, were evaluated by cross-validation using anchored data, as described by Megjhani et al. [24]. The data modeling was performed using Python 3.10.9, scikit-learn version 1.7.0, SciPy 1.15.3, NumPy 1.26.4, XGBoost 3.0.4, and LightGBM 4.6.0.

### 2.5. Real-Time Computation of HRV Features

HRV describes the variations in the interbeat intervals (IBIs) as measured by the distance between consecutive R-peaks in an ECG [12]. An HRV algorithm for detecting R-peaks from ECG signals and computing time- and frequency-domain (FD) indices was developed.

R-peak detection: The original Hamilton algorithm [25] could be improved by modifying the rectification step of the algorithm using a Morlet wavelet instead of a boxcar filter. The original Hamilton algorithm first band-pass filters the ECG signal to suppress DC drifts and high-frequency noise, then differentiates the signal to weigh the steep increases and decreases in the voltage at the flanks of the R-peak, and finally takes the absolute value followed by a boxcar filter to generate a processed signal in which peaks are subsequently located [25]. This approach works well, except when the two flanks of the R-peak are very similarly pronounced, in which case two sub-peaks are generated that can introduce jitter during peak location. To circumvent this problem, we first performed a wavelet transformation and then took the absolute value to achieve rectification that does not exhibit any sub-peaks. Outlier detection was performed using a robust, adaptive threshold method. For each R-peak amplitude, a 3 min centered rolling median was computed to establish a local baseline. Peak deviations from this baseline were calculated, and the spread of these deviations was quantified using the median absolute deviation (MAD). Peaks were flagged as outliers if the absolute deviation from the rolling median exceeded the MAD-based spread by a factor of 8, the peak amplitude fell below 1 × 10^−6^, or the peak amplitude exceeded 3000 units. The IBIs were calculated as the time difference between consecutive R-peaks.

Artifact removal: Missing beats were identified by analyzing the temporal variability in the IBIs. For each IBI, the difference from the preceding IBI was calculated and normalized by a 5 min rolling median IBI to account for local heart rate changes. An IBI was flagged as potentially containing a missing beat when the normalized change exceeded 0.7, indicating a sudden increase in the interval duration of more than 70% relative to the local median. This threshold captures instances where an R-peak was likely undetected, resulting in an artificially prolonged interval spanning two cardiac cycles. Segments were not explicitly screened for cardiac arrhythmia.

Based on the generated peak location and IBIs, the following HRV indices were computed on consecutive, non-overlapping 5 min windows. A window was retained only if it contained enough valid interbeat intervals after artifact removal (≥100 for time-domain and ≥200 for frequency-domain indices), and was otherwise treated as missing.

Time-domain HRV computation: Taking the standard definition of HRV features as described in the guidelines from the Task Force of the European Society of Cardiology and the North American Society of Pacing and Electrophysiology as a starting point [12], the most robust version of the time-domain indices was implemented to reduce or eliminate the effect of, for example, ectopic beats.

Frequency-domain HRV computation: For the computation of the frequency-domain indices, we removed outliers based on their distance from the median relative to the 10% and 90% percentile IBIs before computing the frequency-domain HRV indices. The time series of the IBI values in the rolling 5 min windows was linearly interpolated on a time grid with a sampling time of 20 ms. As a second step, the power spectral density was computed using a fast Fourier transform. Heart rate oscillations are divided into ultra-low-frequency (ULF), very-low-frequency (VLF), low-frequency (LF), and high-frequency (HF) bands [12,26].

Based on the generated peak location and IBIs, the following HRV indices were aggregated into 5 min segments to handle noise and short-term variations in the HRV indices (Table 1).

### 2.6. Feature Engineering

In addition to the raw temporal features, we derived several additional features to capture temporal dynamics and clinically meaningful relationships. For all dynamically updated features (HRV, LAB, and BGA), we computed first-order differences to capture hour-to-hour changes, as these rate-of-change features have been shown to improve prediction of clinical deterioration in ICU settings [24,27]. We also computed the deviation from the expanding (cumulative) patient mean to identify departures from a patient’s evolving baseline, following the rationale that trends relative to a patient’s own trajectory are more informative than absolute values [27,28].

For HRV features specifically, we computed rolling means over 3 h windows to smooth short-term fluctuations, as well as rolling median and minimum statistics over 6, 12, 24, and 48 h windows to capture multi-scale temporal patterns. Multi-scale temporal aggregation of HRV has been shown to reveal prognostic patterns not visible in raw hourly measurements [17,18]. Additionally, we fitted a linear trend (slope) over the entire 48 h observation window for three key time-domain features (AvgNN, SDNN, and HRV-TD-RMSSD) to quantify whether heart rate variability was improving or deteriorating over time, as declining HRV trajectories have been associated with impending DCI onset [20]. We further computed four clinically motivated ratio features: SDNN/HRV-TD-RMSSD, which has been investigated as a time-domain surrogate for sympathovagal balance (LF/HF ratio) [29,30]; HRV-TD-RMSSD/AvgNN as a normalized measure of parasympathetic activity relative to heart rate; lactate/pH as a combined marker of metabolic derangement, motivated by evidence that the trajectory of combined metabolic parameters outperforms individual markers in predicting ICU outcomes [31]; and CRP/Leukocytes as an indicator of systemic inflammatory response intensity relative to immune cell count [32].

In total, 325 features are used for the combined model (see Table S1, Supplementary Materials), with 15 static clinical features, 137 laboratory and blood gas features (47 raw and 90 derived), and 173 HRV features (126 raw and 47 derived). The individual modality models use the corresponding feature subsets independently.

### 2.7. Methods for Performance Evaluation of DCI Prediction Based on HRV Alone

ROC AUC values were calculated for all 173 time-domain (TD) and FD HRV features, including their temporal aggregation variants. An ROC AUC value below 0.5 is associated with a negative correlation between the feature and the outcome. To be able to compare the merit of the positively and negatively correlated features, we multiplied the feature values by −1 for the negatively correlated features to get a non-directional ROC AUC value. The discriminative power of each feature was further analyzed by anchoring the data from the patients at the day of DCI occurrence or at day 7.88 for all other patients, based on the median time to DCI occurrence.

#### Methods for Performance Evaluation of DCI Prediction Based on Multimodal Parameters

To assess the predictive value of different data modalities and their combinations, we evaluated seven feature set configurations: (STATIC) using the 15 static clinical variables including demographics and clinical scores, (LAB/BGA) using the 137 features from laboratory and blood gas analysis [11], as well as their temporal aggregates, (HRV) using 173 HRV features, and (COMBINED) the full combination totaling 325 features. Each feature set was evaluated using six model families (Logistic Regression with L1 and L2 regularization, random forest, Extra Trees, HistGradientBoosting, and XGBoost) with hyperparameter optimization via RandomizedSearchCV (n_iter = 50) within a stratified group cross-validation loop, while generalization performance was assessed on the held-out fold of a 5-fold stratified group cross-validation. The best-performing model family was selected based on the highest mean ROC AUC.

### 2.8. Method of Leave-One-Out Simulation

In addition to the anchored ROC analysis, we conducted leave-one-out (LOO) simulations to evaluate the model’s ability to predict DCI in a scenario more closely resembling the clinical setting in which the models would regularly produce new risk assessments. Based on the model selection experiment described above, the best-performing model family was identified per feature set combination based on mean ROC AUC and was used for all subsequent LOO evaluations. Four feature set configurations were evaluated (STATIC, LAB/BGA, HRV, and COMBINED), where, for each patient, the selected model was trained on the 48 h pre-anchor window of all of the remaining patients, then was used to generate hourly risk predictions across the held-out patient’s entire stay (days 0–14). For patients who developed DCI, hourly predictions were considered positive if they fell within the predefined 48 h window preceding DCI onset. Observations acquired after DCI onset were excluded from model training and evaluation, as these time points are no longer clinically relevant for prediction. Patients without DCI contributed observations throughout the corresponding observation period. Further details on the LOO methodology are given in Willms et al. [11].

### 2.9. Statistical Analysis

The Mann–Whitney U test was used for comparing continuous and ordinal variables, and Fisher’s exact test was used for binary variables. Statistical significance was assumed at *p* < 0.05. The effect size was quantified using the ROC AUC value, where a value above 0.5 indicates a positive association with the outcome and below 0.5 indicates a negative association with the outcome. The confidence intervals for the ROC AUC values in the time-dependent ROC analysis were computed via bootstrapping.

### 2.10. Integration into the Live Data Stream

The IT platform ICU Cockpit^®^ allows for the deployment of containerized applications that can either take advantage of the near-real-time high-resolution data streams to realize event-based data processing tasks or access a live database for implementing scheduled or on-demand tasks [22]. HRV-derived indices are updated with every new heartbeat and published back to the patient’s Kafka topic, thereby augmenting the said data stream and making the HRV indices available to other possible downstream algorithms. Moreover, as the new data becomes part of the patient’s regular data stream, all new signals are automatically stored in the live database with no additional changes needed.

## 3. Results

### 3.1. Participants

Of the 339 patients with aSAH included in the initial study, 101 were eligible for the HRV-based analysis. Eligibility required (1) continuous ECG recordings of sufficient quality and duration to compute reliable HRV features, with no gaps in IBIs longer than 2 h within the 48 h observation window preceding the anchor point, and (2) at least one laboratory or blood gas measurement within the same window. The remaining 238 patients were excluded due to insufficient ECG data availability caused by monitoring gaps, patient procedures, or signal artifacts. To assess potential selection bias, we compared the 101 included patients with the 238 excluded patients across all of the baseline characteristics listed in Table 2 (see Table S3). DCI prevalence did not differ (included 34.7% vs. excluded 39.1%, *p* = 0.46). Demographics and comorbidities were similar between the two groups, with a median age of 59 vs. 57 years, female sex 68% vs. 63%, hypertension 40% vs. 36%, cardiovascular disease 18% vs. 18%, and diabetes 8% vs. 5% (all *p* > 0.05). However, the included cohort had higher admission neurological severity (Hunt and Hess median 3 vs. 2, *p* < 0.001; WFNS 4 vs. 2, *p* = 0.002; extracerebral GCS 12 vs. 14, *p* = 0.002; and modified Fisher *p* < 0.001). A plausible explanation is that more severely affected, sedated, and less mobile patients are more likely to have longer, motion-artifact-free ECG data suitable for HRV computation. The ECG-based selection, therefore, did not retain a less severely affected population, and the prevalence of the prediction target was preserved. Within the selected cohort, the HRV data coverage in the 48 h window was high (mean: 99.5%, median: 100.0%, and minimum: 72.9%), with 88 patients having complete 48 h HRV coverage. The laboratory and blood gas values were forward-filled from the most recent available measurement, resulting in near-complete data availability (<0.1% missing values). An overview of patient characteristics is given in Table 2.

### 3.2. Performance of DCI Prediction Based on HRV Alone—Univariate Analysis

**Figure 1:**
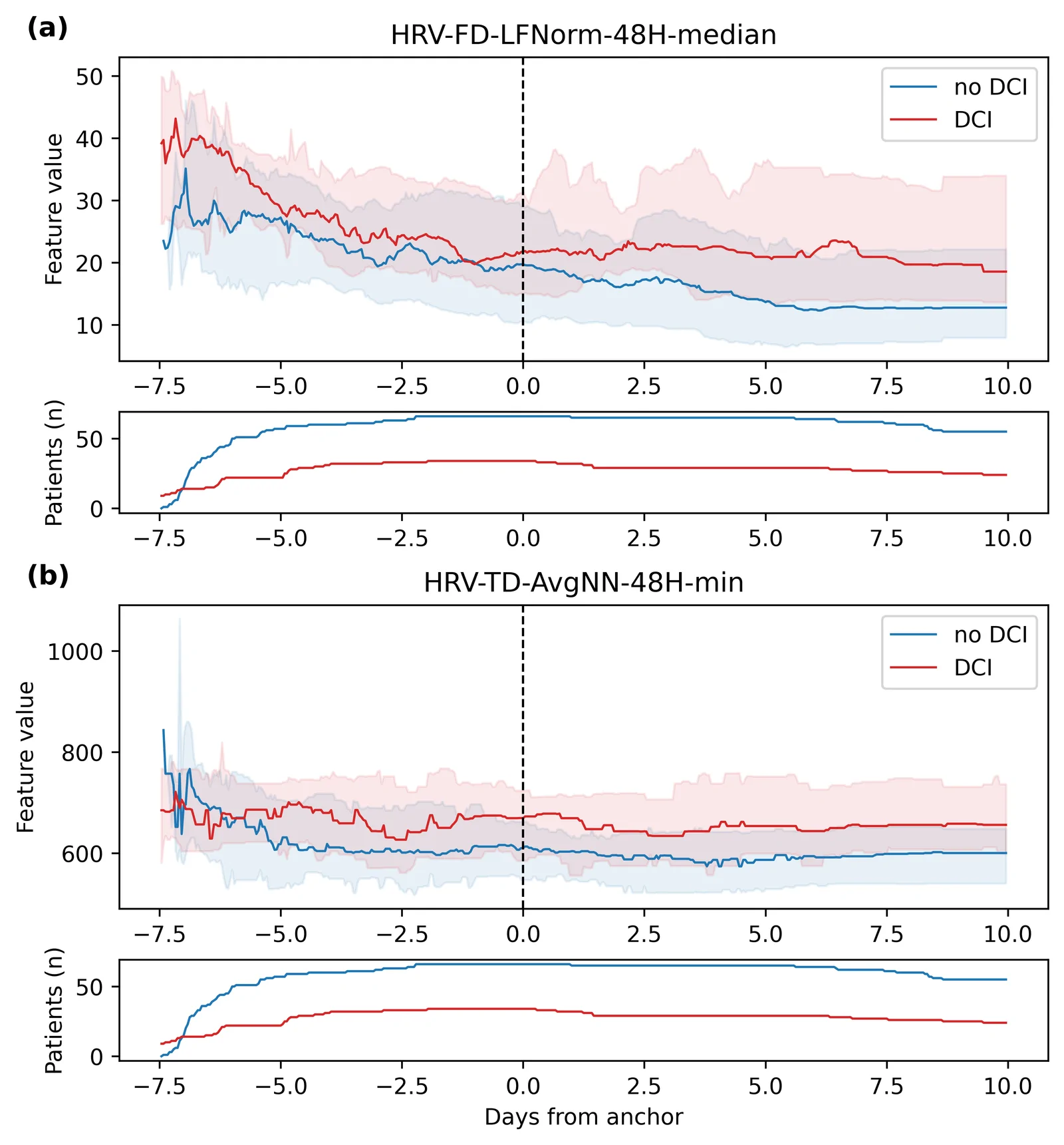
(**a**,**b**): A longitudinal plot of the best-performing HRV features. The feature value scores of patients with and without DCI are plotted in red and blue, respectively. The median scores are plotted as a line together with the interquartile range (colored area).

The frequency-domain parameter LFNorm (normalized power in the low-frequency band) was the best-performing univariate predictor for DCI, with the 48 h median aggregation achieving the highest non-directional ROC AUC (0.64). LFNorm appeared consistently across all aggregation levels within the top 25 features (hourly values: 0.60, and 3 h rolling mean: 0.60, 6 h: 0.61, 12 h: 0.60, 24 h: 0.61, and 48 h: 0.64), indicating robust discriminative value regardless of temporal resolution. The time-domain parameter AvgNN (mean of NN intervals) ranked second overall, with its 48 h minimum and 24 h minimum aggregations both reaching 0.63. Notably, the minimum aggregation outperformed the median for AvgNN across all time windows, suggesting that transient drops in the mean heart rate interval are more predictive of DCI than sustained levels. HFNorm (normalized power in the high-frequency band) complemented LFNorm among the top features, with values between 0.57 and 0.59 across aggregation levels. All top 25 features reached statistical significance (*p* < 0.005). Predictive values for the 25 best-performing features across aggregation levels are given in Table 3 (for the performance of all HRV features, see Table S2 in the Supplementary Materials). The exemplary data for the HRV features HRV-FD-VLF and HRV-FD-HFNorm-6H-median are shown in Figure 1a,b.

#### Performance of DCI Prediction Based on Multimodal Parameters

Among the static models, hyperparameter tuning improved performance, with Extra Trees achieving the best result (ROC AUC: 0.569 ± 0.104), with a maximum tree depth of 1 and a maximum feature fraction of 0.11. For models trained on LAB/BGA features, HistGradientBoosting achieved the best performance (ROC AUC: 0.626 ± 0.087), with a maximum depth of 128, L2 regularization of 2.26, and a learning rate of 0.12. For the HRV feature set, HistGradientBoosting with PCA dimensionality reduction achieved the best result (ROC AUC: 0.601 ± 0.066), using 13 principal components. The need for PCA dimensionality reduction suggests that the high-dimensional HRV feature space contains correlated information that benefits from linear compression before model training. While LAB/BGA features achieved a higher cross-validation score than HRV features alone, the HRV model exhibited lower variance across folds, suggesting more consistent predictive performance. Combining all of the feature sets (STATIC, LAB/BGA, and HRV), random forest achieved the highest overall performance (ROC AUC: 0.68 ± 0.06), with a maximum tree depth of 4 and a maximum feature fraction of 0.11. The combined model outperformed all individual feature set models (STATIC: ROC AUC 0.57 ± 0.10; for LAB/BGA: ROC AUC 0.63 ± 0.09; and HRV: ROC AUC 0.60 ± 0.07), indicating complementary predictive value across modalities.

LAB/BGA features achieved a higher ROC AUC than HRV features alone. The higher dimensionality of the HRV feature space (173 features) required PCA dimensionality reduction to achieve optimal performance, suggesting substantial redundancy among the raw and rolling-window HRV features. In contrast, the LAB/BGA feature set (137 features) could be used directly without dimensionality reduction, which may contribute to its higher individual cross-validation performance. When all modalities were combined, HRV contributed the largest share of feature importance in the best COMBINED model (55.8%, see Supplementary Figure S1). Because this type of importance can be unreliable when features are numerous and correlated, we checked whether the finding was stable (see Supplementary Figure S2). Across the five cross-validation folds, HRV consistently contributed the most and ranked first in every fold. As a complementary method not distorted by correlation, we then reassessed feature importance by removing each modality in turn and recorded how much model performance dropped. Removing HRV lowered the AUC by 0.111, more than twice the drop for LAB/BGA (0.056) and far above static features. Thus, HRV accounted for roughly two-thirds of the total permutation importance. Both methods agreed that HRV is the dominant modality. The most influential features were HRV trajectory measures (24 h and 48 h minima for HRV-TD-AvgNN, HRV-FD-LFNorm, HRV-FD-HFNorm, and patient-level HRV slopes).

We used the best hyperparameter-optimized models per feature set for the subsequent LOO analysis, as their model parameters were learned from the data rather than using arbitrary default settings.

### 3.3. Leave-One-Out Simulation

For the LOO evaluation, each feature set was paired with its best-performing model from the preceding hyperparameter optimization. Using per-modality optimized models ensures that each feature set is evaluated at its full predictive potential, reflecting a realistic clinical deployment scenario in which model architectures are selected independently for each data source.

**Figure 2:**
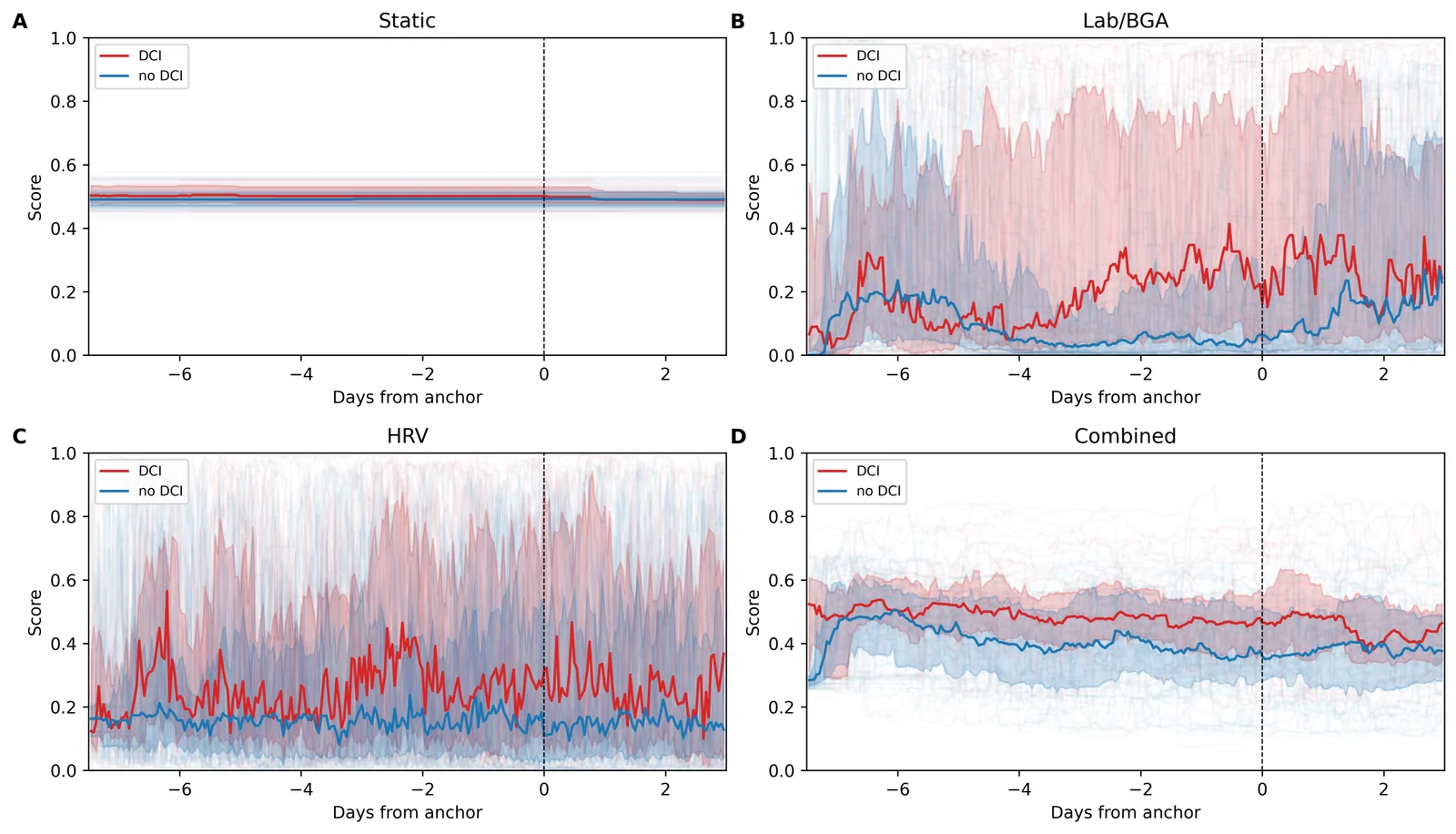
(**A**–**D**): Anchored score trajectories for the different feature sets. Panels (**A**–**D**) summarize the leave-one-out scores for the STATIC, LAB/BGA, HRV, and COMBINED models, respectively. The median scores are plotted as a line together with the interquartile range (colored area). In the background, thin, partially transparent lines indicate the scores of individual patients.

Figure 2 shows the anchored score trajectories for each feature set. The STATIC model (A) showed no meaningful separation between DCI and non-DCI groups despite having individual static features achieving meaningful unimodal discrimination (e.g., notably BNI with an ROC AUC of 0.619). This is likely due to the constrained model’s capacity to leverage complementary information across the 15 static features with only ~100 training patients. The LAB/BGA model (B) showed the most pronounced temporal response, with DCI+ scores rising sharply from day −4 onward and reaching peak separation at day −1, while DCI scores remained consistently low throughout. Notably, LAB/BGA scores showed no separation before day −5, indicating that laboratory markers only become discriminative in close temporal proximity to DCI onset. The HRV model (C) showed an overall positive but variable separation between DCI+ and DCI- trajectories. Brief spikes in separation appeared around day −6.5 and day −2.5, interspersed with periods of weak separation, particularly between day −6 and day −4. The interquartile ranges of DCI+ and DCI- scores overlapped substantially at all time points, reflecting the high variability inherent in hourly HRV-based predictions. The combined model maintained more stable separation across the observation window, smoothing the intermittent signals of the individual modalities.

**Figure 3:**
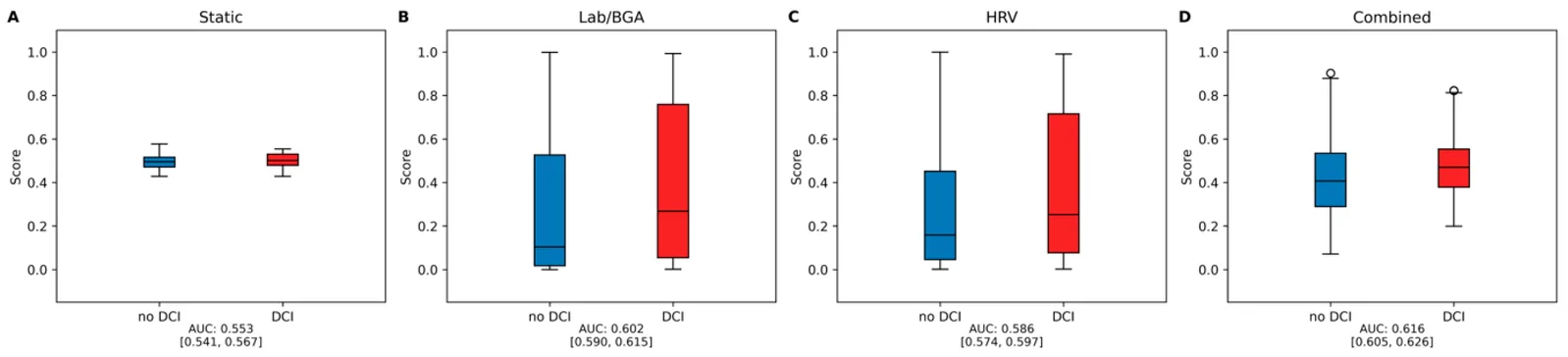
(**A**–**D**): Overall ROC AUCs for the different feature subsets: A comparison of the model output scores for the 48 h leading up to DCI. Boxplots of the model output scores for the different models. The scores were grouped according to whether a DCI occurred in the subsequent 48 h. Only scores up to the first DCI were considered, and patients were removed from the risk set after DCI occurrence. The effect size was measured and is shown as the ROC AUC.

These temporal patterns are reflected in the overall prediction performance (Figure 3). For predicting DCI within 48 h (day 2 onward), the COMBINED model achieved the highest ROC AUC (0.616 [0.604, 0.629]), followed by LAB/BGA (0.602 [0.587, 0.616]) and HRV (0.586 [0.571, 0.599]). The STATIC model (0.553 [0.539, 0.570]) performed near chance level.

**Figure 4:**
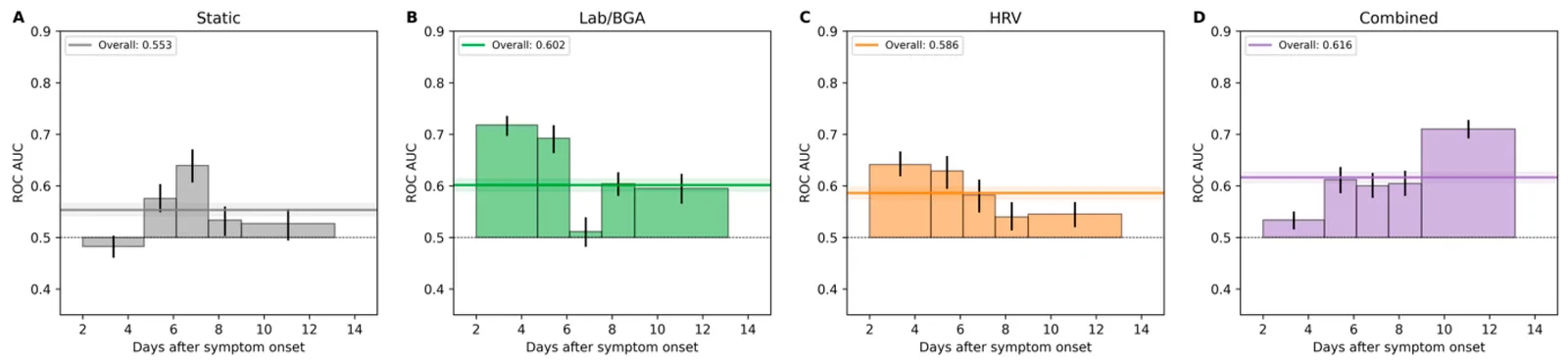
(**A**–**D**): The time-dependent ROC AUC analysis across the different feature subsets and intervals. Panels (**A**–**C**) show the ROC AUC values computed for equal-event intervals: (i) day 2 until day 4.7, (ii) day 4.7 until day 6.1, (iii) day 6.1 until day 7.5, (iv) day 7.5 until day 9, and (v) day 9 until day 13.1 after symptom onset. The 95% confidence intervals are depicted by the vertical lines at the tips of the bars.

Time-dependent analysis (Figure 4) revealed that the predictive value of different feature sets varies substantially over the clinical course. LAB/BGA features dominated the early phase (days 2.0–5.6), achieving the highest interval ROC AUC of 0.718 (days 2.0–4.7) and 0.692 (days 4.7–6.1), consistent with the sharp score separation observed in the trajectory analysis during this period. HRV was the best-performing individual model in the earliest window (days 2.2–3.6, a sliding-window AUC up to 0.683 at day 3), a period where LAB/BGA had not yet developed a discriminative signal. In the mid-phase (days 6.1–7.5), LAB/BGA performance dropped to near chance level (0.511), while the STATIC model briefly achieved the highest discrimination (0.640), suggesting that the baseline clinical severity becomes temporarily more relevant than dynamic markers during this transitional period. The COMBINED model provided the most consistent performance across the full observation period, dominating from day 5.6 onward through most of the remaining stay (days 8.7–13.0), and achieving the highest late-phase interval AUC (0.715, days 9.0–14.0). Consistent with this time dependence, the incremental discrimination of the COMBINED model over LAB/BGA was concentrated in the late phase rather than uniform (Figure S3). In the early interval (days 2.0–4.7), where LAB/BGA already discriminated well (AUC 0.718), adding HRV did not improve performance (ΔAUC −0.18). In the late interval (days 9.0–13.1), the COMBINED model improved discrimination over LAB/BGA (AUC 0.568 → 0.681; ΔAUC +0.11 [−0.02, +0.27]), with a positive integrated discrimination improvement (IDI +0.03). This temporal complementarity supports the use of modality-specific models that can be selected or combined based on the clinical time point and data availability.

Thresholds were computed that achieve a sensitivity level of 80%. Table 4 presents these thresholds along with the corresponding specificity for each interval. The thresholds depend on the considered time intervals and increase together with the corresponding ROC AUC value.

### 3.4. Implementation into Live Data Stream

**Figure 5:**
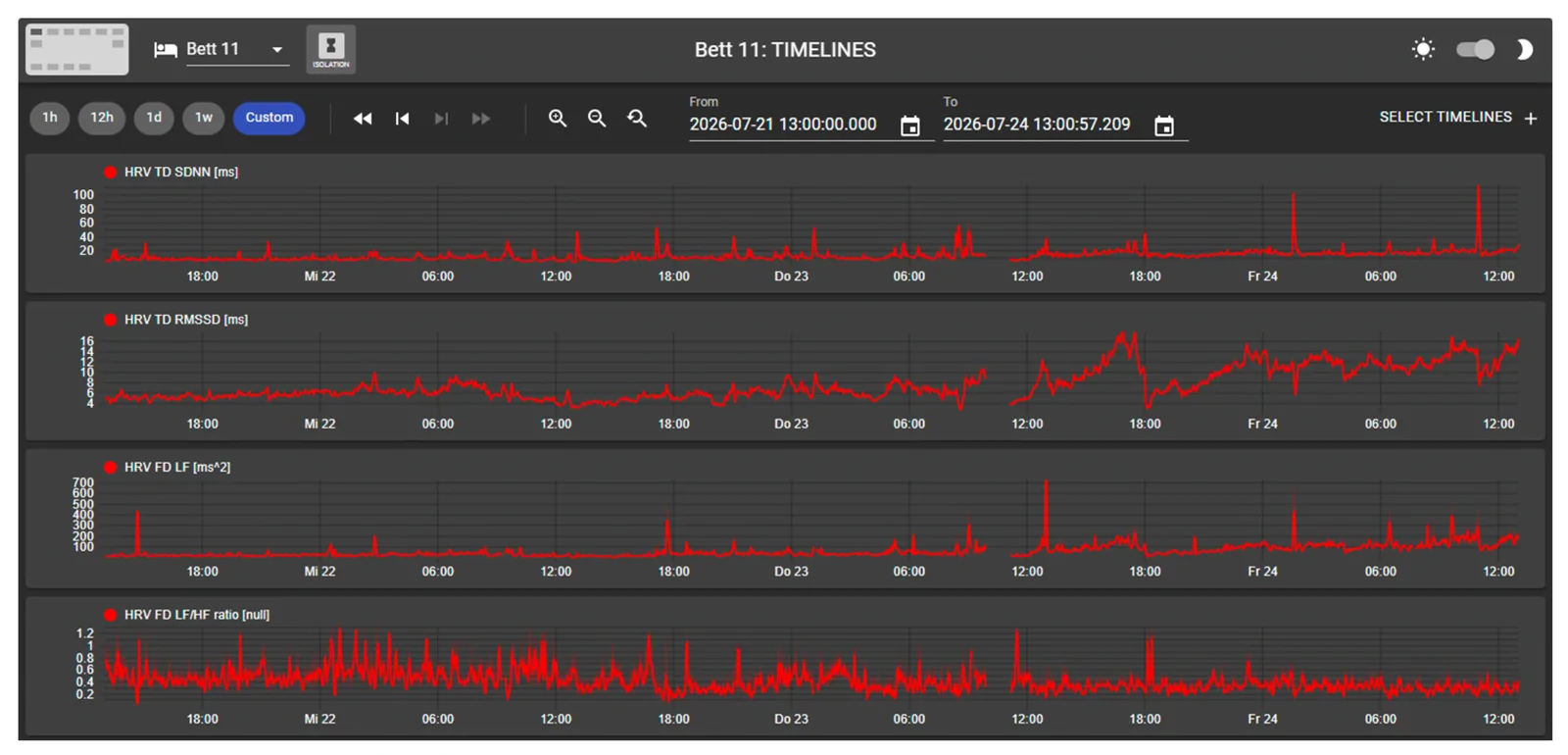
A timeline view of the ICU Cockpit^®^ Dashboard with parameters reflecting HRV (HRV-TD-SDNN, the standard deviation of interbeat intervals; HRV-TD-RMSSD, the root mean square of the successive RR interval differences; HRV-FD-LF, the absolute power in the low-frequency band; and HRV-FD-LF/HF ratio, the ratio of power in the low-frequency band to the high-frequency band).

The set of HRV features was integrated into the ICU Cockpit^®^ as a live stream processing algorithm. Figure 5 displays several HRV features, which were computed dynamically based on a patient’s ECG data and subsequently stored in a database.

## 4. Discussion

### 4.1. Principal Findings

To our knowledge, this study is the first to investigate a data-driven approach for the longitudinal prediction of DCI in patients with aSAH by integrating HRV-derived features with multimodal physiological data. The proposed framework demonstrates the feasibility of using continuously acquired bedside data to support dynamic DCI risk assessment and represents a novel approach to clinical decision support in neurocritical care.

Among the individual predictors, the frequency-domain parameter HRV-FD-LFNorm, summarized as the median over the preceding 48 h, showed the highest unimodal discriminative performance. The integration of HRV-derived features with laboratory and blood gas parameters further improved predictive discrimination, with the multimodal model yielding the best overall results in both cross-validation and leave-one-out evaluation. These findings suggest that HRV captures complementary physiological information beyond routinely available clinical parameters and may contribute to improved longitudinal DCI risk stratification.

### 4.2. Comparison with Previous Studies

Several studies have investigated multimodal or HRV-based approaches for DCI prediction. However, direct comparison with the present study is limited by differences in study design, patient populations, endpoint definitions, available predictors, prediction horizons, and evaluation methods.

Megjhani et al. developed a multimodal model incorporating demographic characteristics and continuously measured vital signs, including heart rate, blood pressure, respiratory rate, and oxygen saturation, and reported an ROC AUC of 0.83, using an ensemble approach based on data from the seven days preceding the anchor time point [24]. Their model included continuously acquired vital-sign variables that were not available in the present analysis, which instead focused on HRV-derived, laboratory, and blood gas features.

Zarrin et al. used cerebral vasospasm requiring intra-arterial verapamil treatment as the prediction endpoint and reported an AUC of 0.88 more than one week before the event, using a gradient boosting model [33]. However, this endpoint, which occurred in 7.1% of the cohort, differs clinically from DCI and, therefore, limits direct comparison with the present findings.

Schmidt et al. developed models for the combined prediction of infection and DCI in 236 patients with aSAH using bootstrap aggregation and cost-sensitive meta-classifiers [20]. The highest ROC AUC was 0.83 for a random forest model combining clinical and HRV-derived predictors 24 h before the event. Similar to the present study, VLF power and related measures were among the most relevant HRV features. The authors concluded, however, that HRV monitoring alone was insufficient to distinguish infectious from ischemic complications.

Bjerkne Wenneberg et al. investigated HRV as a univariable predictor in 55 patients with aSAH and found a higher LF/HF ratio over time among patients who developed DCI than among those who did not [17]. Because HRV was assessed intermittently three times per day, no significant HRV changes immediately preceding DCI were identified. A subsequent analysis of the same cohort applied random forest models to examine HRV patterns in greater depth [18].

Although the present study and the work of Willms et al. were both based on data acquired using the ICU Cockpit^®^ infrastructure, direct comparison remains limited because the ECG quality requirements applied in the present analysis resulted in the inclusion of a smaller and partially different subset of patients [11].

Overall, previous studies support the potential value of HRV-derived and multimodal physiological information for DCI prediction. At the same time, the substantial heterogeneity in patient populations, outcome definitions, predictor availability, prediction horizons, and validation strategies limits direct comparison of reported model performance across studies.

### 4.3. Interpretation

Beyond comparison with previous studies, our findings have several important methodological and clinical implications. In contrast to earlier studies, our model integrates HRV-derived features with laboratory and blood gas parameters to support longitudinal bedside prediction of DCI. Importantly, the primary objective of this work was not to maximize predictive performance but to evaluate the incremental value of HRV-derived features within a multimodal prediction framework.

Furthermore, the LOO evaluation covered up to 14 days after symptom onset, representing a substantially broader temporal scope than the predefined prediction windows used in most comparable studies. This provides a more comprehensive and clinically realistic, albeit more challenging, assessment of predictive performance across different phases of the disease course. Consistent with this, our time-resolved analyses demonstrate that predictive performance varies considerably over time, with different physiological modalities contributing at different stages. These findings suggest that a single aggregated performance metric may not fully reflect the performance characteristics of multimodal prediction systems throughout the clinical course.

Finally, direct numerical comparison of ROC AUC values with previously published DCI prediction studies is limited by fundamental differences in evaluation methodology. Unlike most previous studies, which have formulated DCI prediction as a single patient-level outcome, we adopted a longitudinal row-level evaluation, following Megjhani et al. [24] and Willms et al. [11], generating hourly risk predictions and labeling each time point according to whether it fell within the predefined 48 h prediction window preceding DCI onset. This evaluation strategy represents a more conservative assessment of predictive performance, as it avoids smoothing effects introduced by patient-level aggregation and more closely reflects the intended application in continuous bedside monitoring. Therefore, direct comparison of predictive performance across studies should be interpreted within this methodological context.

### 4.4. Limitations

Several limitations should be considered when interpreting the present findings. First, the requirement for continuous ECG recordings of sufficient quality for HRV computation reduced the eligible cohort to 101 patients, introducing a selection bias and reducing the available training data. Although this constraint was necessary to address the central research question of HRV-based prediction, it inevitably reduced the study population available for model development. Given the relatively small cohort and the limited number of DCI events relative to the number of candidate features, overfitting cannot be completely excluded despite the use of grouped cross-validation, hyperparameter optimization, and leave-one-out evaluation to improve model robustness. The main reasons for exclusion were technical and logistical issues related to continuous ECG data acquisition, including missing device registration at ICU admission, patient transfers for diagnostic procedures, and intermittent data loss due to network-related problems. These challenges highlight the practical difficulties of implementing high-resolution physiological data pipelines in routine clinical environments. Consequently, the observed predictive performance should be interpreted in light of the limited cohort size and the challenges inherent to longitudinal prediction.

Beyond these cohort-related limitations, HRV is influenced by multiple physiological and clinical factors, including changes in posture, patient stress and movement, sedative and analgesic medication, vasopressor therapy and other autonomically active drugs, infections, and mechanical ventilation [13]. In patients with aSAH, additional factors such as systemic infections, ventriculitis associated with external ventricular drains, and other critical care complications may further affect autonomic regulation and thereby HRV measures. These potential confounders were not explicitly accounted for in the present analysis and should, therefore, be considered when interpreting the prediction results. Furthermore, circadian variations in autonomic tone were not explicitly modeled. Consequently, part of the predictive information captured by HRV may reflect overall physiological status or treatment-related effects, rather than DCI-specific pathophysiology alone.

ECG signal artifacts and rhythm abnormalities, including missing or ectopic beats and atrial fibrillation, may introduce noise into HRV computation, particularly when interpolation methods are applied for signal correction [26]. Careful assessment of ECG signal quality is therefore recommended when interpreting HRV features and model outputs in a clinical context.

Finally, although the model is based on routinely collected laboratory values and ECG-derived HRV features, suggesting potential applicability across ICU settings, the present findings should be considered preliminary until validated in independent multicenter cohorts. Internal cross-validation and leave-one-out evaluation provide important estimates of model performance but cannot replace external validation across different monitoring systems, treatment protocols, and ICU environments before clinical implementation can be considered. However, such validation is currently challenging, as no publicly available aSAH datasets provide a comparable combination of high-resolution physiological waveform data suitable for HRV analysis together with longitudinal laboratory and clinical measurements. Moreover, differences in patient populations, evolving treatment strategies, and variability in imaging practices across centers may further limit generalizability.

In our institution, imaging was triggered by a standardized clinical deterioration protocol and predefined neuromonitoring criteria rather than routine scheduled screening. While this approach reflects routine clinical practice at our institution, differences in imaging frequency or thresholds for imaging between institutions may introduce ascertainment bias, affect reported DCI incidence, and should, therefore, be considered when interpreting and generalizing model performance. Continuous monitoring of model performance would, therefore, be essential in any future prospective clinical implementation. In addition, implementation of continuous HRV monitoring over several days requires acquisition and processing of large volumes of high-frequency physiological data and, therefore, depends on dedicated technical infrastructure, such as the ICU Cockpit^®^ system.

## 5. Conclusions

The integration of HRV into a multimodal, longitudinal prediction framework has the potential to enhance DCI risk stratification by providing complementary, time-dependent information beyond conventional clinical and laboratory parameters. The present findings further suggest that predictive performance varies over the clinical course, highlighting the importance of time-resolved modeling approaches in neurocritical care. Taken together, these findings support the potential value of HRV as part of multimodal bedside monitoring. However, further methodological refinement and prospective external validation in independent cohorts are required before clinical implementation can be considered.

## Figures and Tables

**Table 1:** Heart rate variability measurements.

Measurement	Definition
IBI	Interbeat interval
NN	Interbeat intervals from which artifacts have been removed
RR	Interbeat intervals between all successive heartbeats
Time domain	
HRV-TD-AvgNN	Mean of NN intervals
HRV-TD-SDNN	Standard deviation of NN intervals
HRV-TD-RMSSD	Root mean square root of successive RR interval differences
HRV-TD-pNN50	Percentage of RR intervals that differ by at least 50 ms
HRV-TD-pNN10p	Percentage of RR intervals that deviate by more than 10%
HRV-TD-triangularIdx	Integral of the density of the RR interval histogram divided by its height
Frequency domain	
HRV-FD-TP	Total power in the frequency domain
HRV-FD-VLF	Absolute power in the very-low-frequency band (0.0033–0.04 Hz)
HRV-FD-LF	Absolute power in the low-frequency band (0.04–0.15 Hz)
HRV-FD-HF	Absolute power in the high-frequency band (0.15–0.4 Hz)
HRV-FD-LFNorm	Normalized power in the low-frequency band
HRV-FD-HFNorm	Normalized power in the high-frequency band
HRF-FD-LFHFRatio	Ratio of power in the low-frequency band to high-frequency band

**Table 2:** Patient characteristics (DCI, delayed cerebral ischemia; GCS, Glasgow Coma Scale; WFNS, World Federation of Neurological Surgeons scale; mFS, modified Fisher scale; and BNI, Barrow Neurological Institute scale).

Total Patients = 101	Total	DCI+ (*n* = 35)	DCI− (*n* = 66)	*p* Value
Age, year; median (IQR)	59.0 (50.0–66.0)	60.0 (51.0–64.5)	58.0 (50.0–68.0)	0.954
Female sex, *n* (%)	32 (31.7)	8 (22.9)	24 (36.4)	0.185
Hypertension, *n* (%)	40 (39.6)	14 (40.0)	26 (39.4)	1.000
Cardiovascular disease, *n* (%)	18 (18.0)	5 (14.3)	13 (20.0)	0.737
Diabetes, *n* (%)	8 (7.9)	1 (2.9)	7 (10.6)	0.256
GCS, median (IQR)	12.0 (6.0–14.0)	9.0 (4.0–14.0)	13.0 (8.2–14.8)	0.110
Hunt and Hess, median (IQR)	3.0 (2.0–4.0)	3.0 (2.5–5.0)	3.0 (2.0–4.0)	0.103
Hunt and Hess, 4–5; *n* (%)	37 (36.6)	16 (45.7)	21 (31.8)	0.196
WFNS, median (IQR)	4.0 (2.0–5.0)	4.0 (2.0–5.0)	3.0 (2.0–4.0)	0.123
WFNS, 4–5; *n* (%)	52 (51.5)	21 (60.0)	31 (47.0)	0.296
mFS, median (IQR)	4.0 (3.0–4.0)	4.0 (3.5–4.0)	4.0 (3.0–4.0)	0.864
mFS, 3–4; *n* (%)	98 (97.0)	34 (97.1)	64 (97.0)	1.000
BNI, median (IQR)	3.0 (3.0–4.0)	4.0 (3.0–4.0)	3.0 (3.0–4.0)	0.009
BNI, 4–5; *n* (%)	50 (49.5)	23 (65.7)	27 (40.9)	0.022

**Table 3:** Top 25 HRV features.

Measurement	ROC AUC
HRV-FD-LFNorm (48 h median)	0.64
HRV-TD-AvgNN (48 h minimum)	0.63
HRV-TD-AvgNN (24 h minimum)	0.63
HRV-FD-LFNorm (48 h minimum)	0.62
HRV-TD-AvgNN (12 h minimum)	0.62
HRV-TD-AvgNN (6 h minimum)	0.62
HRV-FD-LFNorm (24 h median)	0.61
HRV-FD-LFNorm (6 h median)	0.61
HRV-FD-LFNorm (6 h minimum)	0.60
HRV-FD-LFNorm (3 h rolling mean)	0.60
HRV-FD-LFNorm	0.60
HRV-FD-LFNorm (12 h median)	0.60
HRV-FD-LFNorm (24 h minimum)	0.60
HRV-FD-HFNorm (48 h minimum)	0.59
HRV-TD-AvgNN (48 h median)	0.59
HRV-FD-LFNorm (12 h minimum)	0.59
HRV-FD-HFNorm (48 h median)	0.58
HRV-FD-HFNorm (24 h median)	0.58
HRV-TD-AvgNN (24 h median)	0.58
HRV-TD-AvgNN (3 h rolling mean)	0.58
HRV-TD-AvgNN (6 h median)	0.58
HRV-FD-HFNorm (12 h minimum)	0.58
HRV-TD-AvgNN	0.58
HRV-FD-HFNorm (24 h minimum)	0.58
HRV-FD-HFNorm (12 h median)	0.57

**Table 4:** Thresholds and specificity for a sensitivity level of 80%.

Model	Interval	(i)	(ii)	(iii)	(iv)	(v)
Static	ROC AUC	0.48	0.58	0.64	0.53	0.53
	Threshold	0.48	0.48	0.48	0.47	0.46
	Specificity	0.33	0.34	0.37	0.26	0.04
	Precision	0.06	0.12	0.13	0.12	0.04
LAB/BGA	ROC AUC	0.72	0.69	0.51	0.6	0.59
	Threshold	0.17	0.04	0.01	0.04	0.04
	Specificity	0.6	0.46	0.15	0.41	0.28
	Precision	0.09	0.14	0.1	0.15	0.05
HRV	ROC AUC	0.64	0.63	0.58	0.54	0.55
	Threshold	0.11	0.05	0.04	0.05	0.05
	Specificity	0.38	0.25	0.24	0.28	0.29
	Precision	0.06	0.1	0.11	0.12	0.05
Combined	ROC AUC	0.53	0.61	0.6	0.6	0.71
	Threshold	0.38	0.36	0.35	0.32	0.37
	Specificity	0.34	0.31	0.39	0.35	0.5
	Precision	0.06	0.11	0.14	0.14	0.07

## Data Availability

The data presented in this study are available from the corresponding author upon reasonable request, subject to approval by the responsible institutional and ethical authorities. The data are not publicly available due to patient confidentiality and data protection regulations.

## Supplementary Materials

The following supporting information can be downloaded at: https://www.mdpi.com/article/10.3390/bioengineering13080857/s1, Table S1: Full list of features, Table S2: Unimodal performance of HRV features, Table S3: Baseline characteristics of included versus excluded patients, Figure S1: Impurity-based (the mean decrease in impurity) feature importance from the COMBINED model, Figure S2: The robustness of modality-level feature importance in the COMBINED model, and Figure S3: The time-resolved added value of HRV over the LAB/BGA model.
