# Supplementary material for: Heart Rate Variability Integrated into a Longitudinal Multimodal Model to Predict Delayed Cerebral Ischemia After Aneurysmal Subarachnoid Hemorrhage
Source: Bioengineering (Basel). 2026 Jul 25;13(8):857. doi: 10.3390/bioengineering13080857 (PMC13509250; doi:10.3390/bioengineering13080857)

Table S1: Full list of features. The feature set comprises four categories: (1) static features recorded at admission, including demographics (age, sex), comorbidities, and clinical grading scales (GCS, Hunt and Hess, Modified Fisher, WFNS, BNI); (2) laboratory values spanning hematology, coagulation, inflammation markers, liver and kidney function; (3) blood gas analyses capturing acid-base balance, electrolytes, and oxygenation; and (4) heart rate variability (HRV) metrics derived from both the time domain (e.g., SDNN, RMSSD, pNN50, triangular index) and the frequency domain (e.g., total power, VLF/LF/HF band powers, LF/HF ratio). To capture temporal trends, we additionally compute derived features for laboratory, blood gas, and HRV variables, including hourly changes, deviations from the patient-specific expanding mean, short-term rolling means (3h), rolling medians and minima over multiple windows (6h, 12h, 24h, 48h), and linear trends over 48h. Finally, we include ratio features combining physiologically related measurements, namely lactate/pH, CRP/leukocytes, SDNN/RMSSD, and RMSSD/AvgNN.

Name	Group	Description
Sex	Static	Female = 1, Male = 0
Age	Static	Age in years
Cardiovascular disease	Static	History of cardiovascular disease (binary)
Hypertension	Static	History of hypertension (binary)
Diabetes	Static	History of diabetes (binary)
GCS Eye Opening	Static	Glasgow Coma Scale — eye opening component
GCS Motor	Static	Glasgow Coma Scale — motor component
GCS Intubated	Static	Whether patient was intubated (binary)
GCS Extra	Static	Glasgow Coma Scale — total score
GCS Verbal Extra	Static	Glasgow Coma Scale — verbal component
Hunt and Hess	Static	Hunt and Hess grading scale
Modified Fisher Scale	Static	Modified Fisher Scale grade
WFNS	Static	World Federation of Neurosurgical Societies grade
BNI	Static	Barrow Neurological Institute grade
Hydrocephalus	Static	Hydrocephalus at admission (binary)
BGA-Anion_gap	Lab/BGA	Anion gap
BGA-Ca++	Lab/BGA	Calcium (ionized)
BGA-Cl-	Lab/BGA	Chloride
BGA-Glu	Lab/BGA	Glucose
BGA-Hct	Lab/BGA	Hematocrit
BGA-K+	Lab/BGA	Potassium
BGA-Lac	Lab/BGA	Lactate
BGA-Na+	Lab/BGA	Sodium
BGA-SBE	Lab/BGA	Standard base excess
BGA-mOsm	Lab/BGA	Osmolality
BGA-pCO2T	Lab/BGA	Partial pressure CO2
BGA-pHT	Lab/BGA	pH

BGA-pO2T	Lab/BGA	Partial pressure O2
BGA-sO2	Lab/BGA	Oxygen saturation
BGA-tHb	Lab/BGA	Total hemoglobin
Lab-ALT	Lab/BGA	Alanine aminotransferase
Lab-APTT	Lab/BGA	Activated partial thromboplastin time
Lab-AST	Lab/BGA	Aspartate aminotransferase
Lab-BAS	Lab/BGA	Basophils (%)
Lab-BASA	Lab/BGA	Basophils (absolute)
Lab-CK	Lab/BGA	Creatine kinase
Lab-CKDEPI	Lab/BGA	eGFR (CKD-EPI)
Lab-CRP	Lab/BGA	C-reactive protein
Lab-EC	Lab/BGA	Erythrocyte count
Lab-EOS	Lab/BGA	Eosinophils (%)
Lab-EOSA	Lab/BGA	Eosinophils (absolute)
Lab-FBG	Lab/BGA	Fibrinogen
Lab-IL6H	Lab/BGA	Interleukin-6
Lab-KHINR	Lab/BGA	INR
Lab-KREA	Lab/BGA	Creatinine
Lab-LC	Lab/BGA	Leukocyte count
Lab-LYM	Lab/BGA	Lymphocytes (%)
Lab-LYMA	Lab/BGA	Lymphocytes (absolute)
Lab-MCH	Lab/BGA	Mean corpuscular hemoglobin
Lab-MCHC	Lab/BGA	Mean corpuscular hemoglobin concentration
Lab-MCV	Lab/BGA	Mean corpuscular volume
Lab-MG	Lab/BGA	Magnesium
Lab-MON	Lab/BGA	Monocytes (%)
Lab-MONA	Lab/BGA	Monocytes (absolute)
Lab-NEU	Lab/BGA	Neutrophils (%)
Lab-NEUA	Lab/BGA	Neutrophils (absolute)
Lab-PCT	Lab/BGA	Procalcitonin
Lab-QUICK	Lab/BGA	Quick/PT (%)
Lab-TC	Lab/BGA	Thrombocyte count
Lab-TZI	Lab/BGA	Thrombin time
BGA-Anion_gap (Hourly change)	Lab/BGA	Anion gap — Hourly change
BGA-Ca++ (Hourly change)	Lab/BGA	Calcium (ionized) — Hourly change
BGA-Cl- (Hourly change)	Lab/BGA	Chloride — Hourly change
BGA-Glu (Hourly change)	Lab/BGA	Glucose — Hourly change
BGA-Hct (Hourly change)	Lab/BGA	Hematocrit — Hourly change
BGA-K+ (Hourly change)	Lab/BGA	Potassium — Hourly change

BGA-Lac (Hourly change)	Lab/BGA	Lactate — Hourly change
BGA-Na+ (Hourly change)	Lab/BGA	Sodium — Hourly change
BGA-SBE (Hourly change)	Lab/BGA	Standard base excess — Hourly change
BGA-mOsm (Hourly change)	Lab/BGA	Osmolality — Hourly change
BGA-pCO2T (Hourly change)	Lab/BGA	Partial pressure CO2 — Hourly change
BGA-pHT (Hourly change)	Lab/BGA	pH — Hourly change
BGA-pO2T (Hourly change)	Lab/BGA	Partial pressure O2 — Hourly change
BGA-sO2 (Hourly change)	Lab/BGA	Oxygen saturation — Hourly change
BGA-tHb (Hourly change)	Lab/BGA	Total hemoglobin — Hourly change
Lab-ALT (Hourly change)	Lab/BGA	Alanine aminotransferase — Hourly change
Lab-APTT (Hourly change)	Lab/BGA	Activated partial thromboplastin time — Hourly change
Lab-AST (Hourly change)	Lab/BGA	Aspartate aminotransferase — Hourly change
Lab-BAS (Hourly change)	Lab/BGA	Basophils (%) — Hourly change
Lab-BASA (Hourly change)	Lab/BGA	Basophils (absolute) — Hourly change
Lab-CK (Hourly change)	Lab/BGA	Creatine kinase — Hourly change
Lab-CKDEPI (Hourly change)	Lab/BGA	eGFR (CKD-EPI) — Hourly change
Lab-CRP (Hourly change)	Lab/BGA	C-reactive protein — Hourly change
Lab-EC (Hourly change)	Lab/BGA	Erythrocyte count — Hourly change
Lab-EOS (Hourly change)	Lab/BGA	Eosinophils (%) — Hourly change
Lab-EOSA (Hourly change)	Lab/BGA	Eosinophils (absolute) — Hourly change
Lab-FBG (Hourly change)	Lab/BGA	Fibrinogen — Hourly change
Lab-IL6H (Hourly change)	Lab/BGA	Interleukin-6 — Hourly change
Lab-KHINR (Hourly change)	Lab/BGA	INR — Hourly change
Lab-KREA (Hourly change)	Lab/BGA	Creatinine — Hourly change
Lab-LC (Hourly change)	Lab/BGA	Leukocyte count — Hourly change

Lab-LYM (Hourly change)	Lab/BGA	Lymphocytes (%) — Hourly change
Lab-LYMA (Hourly change)	Lab/BGA	Lymphocytes (absolute) — Hourly change
Lab-MCH (Hourly change)	Lab/BGA	Mean corpuscular hemoglobin — Hourly change
Lab-MCHC (Hourly change)	Lab/BGA	Mean corpuscular hemoglobin concentration — Hourly change
Lab-MCV (Hourly change)	Lab/BGA	Mean corpuscular volume — Hourly change
Lab-MG (Hourly change)	Lab/BGA	Magnesium — Hourly change
Lab-MON (Hourly change)	Lab/BGA	Monocytes (%) — Hourly change
Lab-MONA (Hourly change)	Lab/BGA	Monocytes (absolute) — Hourly change
Lab-NEU (Hourly change)	Lab/BGA	Neutrophils (%) — Hourly change
Lab-NEUA (Hourly change)	Lab/BGA	Neutrophils (absolute) — Hourly change
Lab-PCT (Hourly change)	Lab/BGA	Procalcitonin — Hourly change
Lab-QUICK (Hourly change)	Lab/BGA	Quick/PT (%) — Hourly change
Lab-TC (Hourly change)	Lab/BGA	Thrombocyte count — Hourly change
Lab-TZI (Hourly change)	Lab/BGA	Thrombin time — Hourly change
BGA-Anion_gap (Deviation from expanding mean)	Lab/BGA	Anion gap — Deviation from expanding mean
BGA-Ca++ (Deviation from expanding mean)	Lab/BGA	Calcium (ionized) — Deviation from expanding mean
BGA-Cl- (Deviation from expanding mean)	Lab/BGA	Chloride — Deviation from expanding mean
BGA-Glu (Deviation from expanding mean)	Lab/BGA	Glucose — Deviation from expanding mean
BGA-Hct (Deviation from expanding mean)	Lab/BGA	Hematocrit — Deviation from expanding mean
BGA-K+ (Deviation from expanding mean)	Lab/BGA	Potassium — Deviation from expanding mean
BGA-Lac (Deviation from expanding mean)	Lab/BGA	Lactate — Deviation from expanding mean

BGA-Na+ (Deviation from expanding mean)	Lab/BGA	Sodium — Deviation from expanding mean
BGA-SBE (Deviation from expanding mean)	Lab/BGA	Standard base excess — Deviation from expanding mean
BGA-mOsm (Deviation from expanding mean)	Lab/BGA	Osmolality — Deviation from expanding mean
BGA-pCO2T (Deviation from expanding mean)	Lab/BGA	Partial pressure CO2 — Deviation from expanding mean
BGA-pHT (Deviation from expanding mean)	Lab/BGA	pH — Deviation from expanding mean
BGA-pO2T (Deviation from expanding mean)	Lab/BGA	Partial pressure O2 — Deviation from expanding mean
BGA-sO2 (Deviation from expanding mean)	Lab/BGA	Oxygen saturation — Deviation from expanding mean
BGA-tHb (Deviation from expanding mean)	Lab/BGA	Total hemoglobin — Deviation from expanding mean
Lab-ALT (Deviation from expanding mean)	Lab/BGA	Alanine aminotransferase — Deviation from expanding mean
Lab-APTT (Deviation from expanding mean)	Lab/BGA	Activated partial thromboplastin time — Deviation from expanding mean
Lab-AST (Deviation from expanding mean)	Lab/BGA	Aspartate aminotransferase — Deviation from expanding mean
Lab-BAS (Deviation from expanding mean)	Lab/BGA	Basophils (%) — Deviation from expanding mean
Lab-BASA (Deviation from expanding mean)	Lab/BGA	Basophils (absolute) — Deviation from expanding mean
Lab-CK (Deviation from expanding mean)	Lab/BGA	Creatine kinase — Deviation from expanding mean
Lab-CKDEPI (Deviation from expanding mean)	Lab/BGA	eGFR (CKD-EPI) — Deviation from expanding mean
Lab-CRP (Deviation from expanding mean)	Lab/BGA	C-reactive protein — Deviation from expanding mean
Lab-EC (Deviation from expanding mean)	Lab/BGA	Erythrocyte count — Deviation from expanding mean

Lab-EOS (Deviation from expanding mean)	Lab/BGA	Eosinophils (%) — Deviation from expanding mean
Lab-EOSA (Deviation from expanding mean)	Lab/BGA	Eosinophils (absolute) — Deviation from expanding mean
Lab-FBG (Deviation from expanding mean)	Lab/BGA	Fibrinogen — Deviation from expanding mean
Lab-IL6H (Deviation from expanding mean)	Lab/BGA	Interleukin-6 — Deviation from expanding mean
Lab-KHINR (Deviation from expanding mean)	Lab/BGA	INR — Deviation from expanding mean
Lab-KREA (Deviation from expanding mean)	Lab/BGA	Creatinine — Deviation from expanding mean
Lab-LC (Deviation from expanding mean)	Lab/BGA	Leukocyte count — Deviation from expanding mean
Lab-LYM (Deviation from expanding mean)	Lab/BGA	Lymphocytes (%) — Deviation from expanding mean
Lab-LYMA (Deviation from expanding mean)	Lab/BGA	Lymphocytes (absolute) — Deviation from expanding mean
Lab-MCH (Deviation from expanding mean)	Lab/BGA	Mean corpuscular hemoglobin — Deviation from expanding mean
Lab-MCHC (Deviation from expanding mean)	Lab/BGA	Mean corpuscular hemoglobin concentration — Deviation from expanding mean
Lab-MCV (Deviation from expanding mean)	Lab/BGA	Mean corpuscular volume — Deviation from expanding mean
Lab-MG (Deviation from expanding mean)	Lab/BGA	Magnesium — Deviation from expanding mean
Lab-MON (Deviation from expanding mean)	Lab/BGA	Monocytes (%) — Deviation from expanding mean
Lab-MONA (Deviation from expanding mean)	Lab/BGA	Monocytes (absolute) — Deviation from expanding mean
Lab-NEU (Deviation from expanding mean)	Lab/BGA	Neutrophils (%) — Deviation from expanding mean
Lab-NEUA (Deviation from expanding mean)	Lab/BGA	Neutrophils (absolute) — Deviation from expanding mean

Lab-PCT (Deviation from expanding mean)	Lab/BGA	Procalcitonin — Deviation from expanding mean
Lab-QUICK (Deviation from expanding mean)	Lab/BGA	Quick/PT (%) — Deviation from expanding mean
Lab-TC (Deviation from expanding mean)	Lab/BGA	Thrombocyte count — Deviation from expanding mean
Lab-TZI (Deviation from expanding mean)	Lab/BGA	Thrombin time — Deviation from expanding mean
BGA-Lactate/pH	Lab/BGA	Ratio of lactate to pH
Lab-CRP/Leukocytes	Lab/BGA	Ratio of CRP to leukocyte count
HRV-TD-AvgNN	HRV	Mean of NN intervals
HRV-TD-SDNN	HRV	Standard deviation of NN intervals
HRV-TD-RMSSD	HRV	Root mean square of successive RR interval differences
HRV-TD-SDSD	HRV	Standard deviation of successive RR interval differences
HRV-TD-pNN50	HRV	Percentage of RR intervals differing by at least 50ms
HRV-TD-ppNN10	HRV	Percentage of RR intervals deviating by more than 10%
HRV-TD-TriangularIdx	HRV	Integral of RR interval histogram density divided by its height
HRV-TD-AvgNN (6h median)	HRV	Mean of NN intervals — 6h median
HRV-TD-AvgNN (6h minimum)	HRV	Mean of NN intervals — 6h minimum
HRV-TD-AvgNN (12h median)	HRV	Mean of NN intervals — 12h median
HRV-TD-AvgNN (12h minimum)	HRV	Mean of NN intervals — 12h minimum
HRV-TD-AvgNN (24h median)	HRV	Mean of NN intervals — 24h median
HRV-TD-AvgNN (24h minimum)	HRV	Mean of NN intervals — 24h minimum
HRV-TD-AvgNN (48h median)	HRV	Mean of NN intervals — 48h median
HRV-TD-AvgNN (48h minimum)	HRV	Mean of NN intervals — 48h minimum
HRV-TD-SDNN (6h median)	HRV	Standard deviation of NN intervals — 6h median
HRV-TD-SDNN (6h minimum)	HRV	Standard deviation of NN intervals — 6h minimum
HRV-TD-SDNN (12h median)	HRV	Standard deviation of NN intervals — 12h median
HRV-TD-SDNN (12h minimum)	HRV	Standard deviation of NN intervals — 12h minimum
HRV-TD-SDNN (24h median)	HRV	Standard deviation of NN intervals — 24h median

HRV-TD-SDNN (24h minimum)	HRV	Standard deviation of NN intervals — 24h minimum
HRV-TD-SDNN (48h median)	HRV	Standard deviation of NN intervals — 48h median
HRV-TD-SDNN (48h minimum)	HRV	Standard deviation of NN intervals — 48h minimum
HRV-TD-RMSSD (6h median)	HRV	Root mean square of successive RR interval differences — 6h median
HRV-TD-RMSSD (6h minimum)	HRV	Root mean square of successive RR interval differences — 6h minimum
HRV-TD-RMSSD (12h median)	HRV	Root mean square of successive RR interval differences — 12h median
HRV-TD-RMSSD (12h minimum)	HRV	Root mean square of successive RR interval differences — 12h minimum
HRV-TD-RMSSD (24h median)	HRV	Root mean square of successive RR interval differences — 24h median
HRV-TD-RMSSD (24h minimum)	HRV	Root mean square of successive RR interval differences — 24h minimum
HRV-TD-RMSSD (48h median)	HRV	Root mean square of successive RR interval differences — 48h median
HRV-TD-RMSSD (48h minimum)	HRV	Root mean square of successive RR interval differences — 48h minimum
HRV-TD-SDSD (6h median)	HRV	Standard deviation of successive RR interval differences — 6h median
HRV-TD-SDSD (6h minimum)	HRV	Standard deviation of successive RR interval differences — 6h minimum
HRV-TD-SDSD (12h median)	HRV	Standard deviation of successive RR interval differences — 12h median
HRV-TD-SDSD (12h minimum)	HRV	Standard deviation of successive RR interval differences — 12h minimum
HRV-TD-SDSD (24h median)	HRV	Standard deviation of successive RR interval differences — 24h median
HRV-TD-SDSD (24h minimum)	HRV	Standard deviation of successive RR interval differences — 24h minimum
HRV-TD-SDSD (48h median)	HRV	Standard deviation of successive RR interval differences — 48h median
HRV-TD-SDSD (48h minimum)	HRV	Standard deviation of successive RR interval differences — 48h minimum
HRV-TD-pNN50 (6h median)	HRV	Percentage of RR intervals differing by at least 50ms — 6h median
HRV-TD-pNN50 (6h minimum)	HRV	Percentage of RR intervals differing by at least 50ms — 6h minimum
HRV-TD-pNN50 (12h median)	HRV	Percentage of RR intervals differing by at least 50ms — 12h median
HRV-TD-pNN50 (12h minimum)	HRV	Percentage of RR intervals differing by at least 50ms — 12h minimum
HRV-TD-pNN50 (24h median)	HRV	Percentage of RR intervals differing by at least 50ms — 24h median
HRV-TD-pNN50 (24h minimum)	HRV	Percentage of RR intervals differing by at least 50ms — 24h minimum

HRV-TD-pNN50 (48h median)	HRV	Percentage of RR intervals differing by at least 50ms — 48h median
HRV-TD-pNN50 (48h minimum)	HRV	Percentage of RR intervals differing by at least 50ms — 48h minimum
HRV-TD-ppNN10 (6h median)	HRV	Percentage of RR intervals deviating by more than 10% — 6h median
HRV-TD-ppNN10 (6h minimum)	HRV	Percentage of RR intervals deviating by more than 10% — 6h minimum
HRV-TD-ppNN10 (12h median)	HRV	Percentage of RR intervals deviating by more than 10% — 12h median
HRV-TD-ppNN10 (12h minimum)	HRV	Percentage of RR intervals deviating by more than 10% — 12h minimum
HRV-TD-ppNN10 (24h median)	HRV	Percentage of RR intervals deviating by more than 10% — 24h median
HRV-TD-ppNN10 (24h minimum)	HRV	Percentage of RR intervals deviating by more than 10% — 24h minimum
HRV-TD-ppNN10 (48h median)	HRV	Percentage of RR intervals deviating by more than 10% — 48h median
HRV-TD-ppNN10 (48h minimum)	HRV	Percentage of RR intervals deviating by more than 10% — 48h minimum
HRV-TD-TriangularIdx (6h median)	HRV	Integral of RR interval histogram density divided by its height — 6h median
HRV-TD-TriangularIdx (6h minimum)	HRV	Integral of RR interval histogram density divided by its height — 6h minimum
HRV-TD-TriangularIdx (12h median)	HRV	Integral of RR interval histogram density divided by its height — 12h median
HRV-TD-TriangularIdx (12h minimum)	HRV	Integral of RR interval histogram density divided by its height — 12h minimum
HRV-TD-TriangularIdx (24h median)	HRV	Integral of RR interval histogram density divided by its height — 24h median
HRV-TD-TriangularIdx (24h minimum)	HRV	Integral of RR interval histogram density divided by its height — 24h minimum
HRV-TD-TriangularIdx (48h median)	HRV	Integral of RR interval histogram density divided by its height — 48h median
HRV-TD-TriangularIdx (48h minimum)	HRV	Integral of RR interval histogram density divided by its height — 48h minimum
HRV-FD-TP	HRV	Total power in the frequency domain
HRV-FD-VLF	HRV	Absolute power in very low frequency band (0.0033-0.04 Hz)
HRV-FD-LF	HRV	Absolute power in low frequency band (0.04-0.15 Hz)
HRV-FD-HF	HRV	Absolute power in high frequency band (0.15-0.4 Hz)
HRV-FD-LFNorm	HRV	Normalized power in low frequency band
HRV-FD-HFNorm	HRV	Normalized power in high frequency band
HRV-FD-LFHFRatio	HRV	Ratio of LF to HF power
HRV-FD-TP (6h median)	HRV	Total power in the frequency domain — 6h median
HRV-FD-TP (6h minimum)	HRV	Total power in the frequency domain — 6h minimum
HRV-FD-TP (12h median)	HRV	Total power in the frequency domain — 12h median

HRV-FD-TP (12h minimum)	HRV	Total power in the frequency domain — 12h minimum
HRV-FD-TP (24h median)	HRV	Total power in the frequency domain — 24h median
HRV-FD-TP (24h minimum)	HRV	Total power in the frequency domain — 24h minimum
HRV-FD-TP (48h median)	HRV	Total power in the frequency domain — 48h median
HRV-FD-TP (48h minimum)	HRV	Total power in the frequency domain — 48h minimum
HRV-FD-VLF (6h median)	HRV	Absolute power in very low frequency band (0.0033-0.04 Hz) — 6h median
HRV-FD-VLF (6h minimum)	HRV	Absolute power in very low frequency band (0.0033-0.04 Hz) — 6h minimum
HRV-FD-VLF (12h median)	HRV	Absolute power in very low frequency band (0.0033-0.04 Hz) — 12h median
HRV-FD-VLF (12h minimum)	HRV	Absolute power in very low frequency band (0.0033-0.04 Hz) — 12h minimum
HRV-FD-VLF (24h median)	HRV	Absolute power in very low frequency band (0.0033-0.04 Hz) — 24h median
HRV-FD-VLF (24h minimum)	HRV	Absolute power in very low frequency band (0.0033-0.04 Hz) — 24h minimum
HRV-FD-VLF (48h median)	HRV	Absolute power in very low frequency band (0.0033-0.04 Hz) — 48h median
HRV-FD-VLF (48h minimum)	HRV	Absolute power in very low frequency band (0.0033-0.04 Hz) — 48h minimum
HRV-FD-LF (6h median)	HRV	Absolute power in low frequency band (0.04-0.15 Hz) — 6h median
HRV-FD-LF (6h minimum)	HRV	Absolute power in low frequency band (0.04-0.15 Hz) — 6h minimum
HRV-FD-LF (12h median)	HRV	Absolute power in low frequency band (0.04-0.15 Hz) — 12h median
HRV-FD-LF (12h minimum)	HRV	Absolute power in low frequency band (0.04-0.15 Hz) — 12h minimum
HRV-FD-LF (24h median)	HRV	Absolute power in low frequency band (0.04-0.15 Hz) — 24h median
HRV-FD-LF (24h minimum)	HRV	Absolute power in low frequency band (0.04-0.15 Hz) — 24h minimum
HRV-FD-LF (48h median)	HRV	Absolute power in low frequency band (0.04-0.15 Hz) — 48h median
HRV-FD-LF (48h minimum)	HRV	Absolute power in low frequency band (0.04-0.15 Hz) — 48h minimum
HRV-FD-HF (6h median)	HRV	Absolute power in high frequency band (0.15-0.4 Hz) — 6h median
HRV-FD-HF (6h minimum)	HRV	Absolute power in high frequency band (0.15-0.4 Hz) — 6h minimum
HRV-FD-HF (12h median)	HRV	Absolute power in high frequency band (0.15-0.4 Hz) — 12h median
HRV-FD-HF (12h minimum)	HRV	Absolute power in high frequency band (0.15-0.4 Hz) — 12h minimum

HRV-FD-HF (24h median)	HRV	Absolute power in high frequency band (0.15-0.4 Hz) — 24h median
HRV-FD-HF (24h minimum)	HRV	Absolute power in high frequency band (0.15-0.4 Hz) — 24h minimum
HRV-FD-HF (48h median)	HRV	Absolute power in high frequency band (0.15-0.4 Hz) — 48h median
HRV-FD-HF (48h minimum)	HRV	Absolute power in high frequency band (0.15-0.4 Hz) — 48h minimum
HRV-FD-LFNorm (6h median)	HRV	Normalized power in low frequency band — 6h median
HRV-FD-LFNorm (6h minimum)	HRV	Normalized power in low frequency band — 6h minimum
HRV-FD-LFNorm (12h median)	HRV	Normalized power in low frequency band — 12h median
HRV-FD-LFNorm (12h minimum)	HRV	Normalized power in low frequency band — 12h minimum
HRV-FD-LFNorm (24h median)	HRV	Normalized power in low frequency band — 24h median
HRV-FD-LFNorm (24h minimum)	HRV	Normalized power in low frequency band — 24h minimum
HRV-FD-LFNorm (48h median)	HRV	Normalized power in low frequency band — 48h median
HRV-FD-LFNorm (48h minimum)	HRV	Normalized power in low frequency band — 48h minimum
HRV-FD-HFNorm (6h median)	HRV	Normalized power in high frequency band — 6h median
HRV-FD-HFNorm (6h minimum)	HRV	Normalized power in high frequency band — 6h minimum
HRV-FD-HFNorm (12h median)	HRV	Normalized power in high frequency band — 12h median
HRV-FD-HFNorm (12h minimum)	HRV	Normalized power in high frequency band — 12h minimum
HRV-FD-HFNorm (24h median)	HRV	Normalized power in high frequency band — 24h median
HRV-FD-HFNorm (24h minimum)	HRV	Normalized power in high frequency band — 24h minimum
HRV-FD-HFNorm (48h median)	HRV	Normalized power in high frequency band — 48h median
HRV-FD-HFNorm (48h minimum)	HRV	Normalized power in high frequency band — 48h minimum
HRV-FD-LFHFRatio (6h median)	HRV	Ratio of LF to HF power — 6h median
HRV-FD-LFHFRatio (6h minimum)	HRV	Ratio of LF to HF power — 6h minimum
HRV-FD-LFHFRatio (12h median)	HRV	Ratio of LF to HF power — 12h median
HRV-FD-LFHFRatio (12h minimum)	HRV	Ratio of LF to HF power — 12h minimum
HRV-FD-LFHFRatio (24h median)	HRV	Ratio of LF to HF power — 24h median

HRV-FD-LFHFRatio (24h minimum)	HRV	Ratio of LF to HF power — 24h minimum
HRV-FD-LFHFRatio (48h median)	HRV	Ratio of LF to HF power — 48h median
HRV-FD-LFHFRatio (48h minimum)	HRV	Ratio of LF to HF power — 48h minimum
HRV-TD-AvgNN (Hourly change)	HRV	Mean of NN intervals — Hourly change
HRV-TD-SDNN (Hourly change)	HRV	Standard deviation of NN intervals — Hourly change
HRV-TD-RMSSD (Hourly change)	HRV	Root mean square of successive RR interval differences — Hourly change
HRV-TD-SDSD (Hourly change)	HRV	Standard deviation of successive RR interval differences — Hourly change
HRV-TD-pNN50 (Hourly change)	HRV	Percentage of RR intervals differing by at least 50ms — Hourly change
HRV-TD-ppNN10 (Hourly change)	HRV	Percentage of RR intervals deviating by more than 10% — Hourly change
HRV-TD-TriangularIdx (Hourly change)	HRV	Integral of RR interval histogram density divided by its height — Hourly change
HRV-FD-TP (Hourly change)	HRV	Total power in the frequency domain — Hourly change
HRV-FD-VLF (Hourly change)	HRV	Absolute power in very low frequency band (0.0033-0.04 Hz) — Hourly change
HRV-FD-LF (Hourly change)	HRV	Absolute power in low frequency band (0.04-0.15 Hz) — Hourly change
HRV-FD-HF (Hourly change)	HRV	Absolute power in high frequency band (0.15-0.4 Hz) — Hourly change
HRV-FD-LFNorm (Hourly change)	HRV	Normalized power in low frequency band — Hourly change
HRV-FD-HFNorm (Hourly change)	HRV	Normalized power in high frequency band — Hourly change
HRV-FD-LFHFRatio (Hourly change)	HRV	Ratio of LF to HF power — Hourly change
HRV-TD-AvgNN (Deviation from expanding mean)	HRV	Mean of NN intervals — Deviation from expanding mean
HRV-TD-SDNN (Deviation from expanding mean)	HRV	Standard deviation of NN intervals — Deviation from expanding mean
HRV-TD-RMSSD (Deviation from expanding mean)	HRV	Root mean square of successive RR interval differences — Deviation from expanding mean
HRV-TD-SDSD (Deviation from expanding mean)	HRV	Standard deviation of successive RR interval differences — Deviation from expanding mean
HRV-TD-pNN50 (Deviation from expanding mean)	HRV	Percentage of RR intervals differing by at least 50ms — Deviation from expanding mean

HRV-TD-ppNN10 (Deviation from expanding mean)	HRV	Percentage of RR intervals deviating by more than 10% — Deviation from expanding mean
HRV-TD-TriangularIdx (Deviation from expanding mean)	HRV	Integral of RR interval histogram density divided by its height — Deviation from expanding mean
HRV-FD-TP (Deviation from expanding mean)	HRV	Total power in the frequency domain — Deviation from expanding mean
HRV-FD-VLF (Deviation from expanding mean)	HRV	Absolute power in very low frequency band (0.0033-0.04 Hz) — Deviation from expanding mean
HRV-FD-LF (Deviation from expanding mean)	HRV	Absolute power in low frequency band (0.04-0.15 Hz) — Deviation from expanding mean
HRV-FD-HF (Deviation from expanding mean)	HRV	Absolute power in high frequency band (0.15-0.4 Hz) — Deviation from expanding mean
HRV-FD-LFNorm (Deviation from expanding mean)	HRV	Normalized power in low frequency band — Deviation from expanding mean
HRV-FD-HFNorm (Deviation from expanding mean)	HRV	Normalized power in high frequency band — Deviation from expanding mean
HRV-FD-LFHFRatio (Deviation from expanding mean)	HRV	Ratio of LF to HF power — Deviation from expanding mean
HRV-TD-SDNN/RMSSD	HRV	Ratio of SDNN to RMSSD
HRV-TD-RMSSD/AvgNN	HRV	Ratio of RMSSD to AvgNN
HRV-TD-AvgNN (3h rolling mean)	HRV	Mean of NN intervals — 3h rolling mean
HRV-TD-SDNN (3h rolling mean)	HRV	Standard deviation of NN intervals — 3h rolling mean
HRV-TD-RMSSD (3h rolling mean)	HRV	Root mean square of successive RR interval differences — 3h rolling mean
HRV-TD-SDSD (3h rolling mean)	HRV	Standard deviation of successive RR interval differences — 3h rolling mean
HRV-TD-pNN50 (3h rolling mean)	HRV	Percentage of RR intervals differing by at least 50ms — 3h rolling mean
HRV-TD-ppNN10 (3h rolling mean)	HRV	Percentage of RR intervals deviating by more than 10% — 3h rolling mean
HRV-TD-TriangularIdx (3h rolling mean)	HRV	Integral of RR interval histogram density divided by its height — 3h rolling mean
HRV-FD-TP (3h rolling mean)	HRV	Total power in the frequency domain — 3h rolling mean
HRV-FD-VLF (3h rolling mean)	HRV	Absolute power in very low frequency band (0.0033-0.04 Hz) — 3h rolling mean
HRV-FD-LF (3h rolling mean)	HRV	Absolute power in low frequency band (0.04-0.15 Hz) — 3h rolling mean

HRV-FD-HF (3h rolling mean)	HRV	Absolute power in high frequency band (0.15-0.4 Hz) — 3h rolling mean
HRV-FD-LFNorm (3h rolling mean)	HRV	Normalized power in low frequency band — 3h rolling mean
HRV-FD-HFNorm (3h rolling mean)	HRV	Normalized power in high frequency band — 3h rolling mean
HRV-FD-LFHFRatio (3h rolling mean)	HRV	Ratio of LF to HF power — 3h rolling mean
HRV-TD-AvgNN (Linear trend (48h))	HRV	Mean of NN intervals — Linear trend (48h)
HRV-TD-SDNN (Linear trend (48h))	HRV	Standard deviation of NN intervals — Linear trend (48h)
HRV-TD-RMSSD (Linear trend (48h))	HRV	Root mean square of successive RR interval differences — Linear trend (48h)

Table S2: Unimodal performance of HRV features

Feature Name	ROC AUC	ROC AUC (non-directional)	P value
HRV-FD-LFNorm (48h median)	0,64	0,64	0,00
HRV-TD-AvgNN (48h minimum)	0,63	0,63	0,00
HRV-TD-SDNN/RMSSD ratio	0,37	0,63	0,00
HRV-TD-AvgNN (24h minimum)	0,63	0,63	0,00
HRV-FD-LFNorm (48h minimum)	0,62	0,62	0,00
HRV-TD-AvgNN (12h minimum)	0,62	0,62	0,00
HRV-TD-AvgNN (6h minimum)	0,62	0,62	0,00
HRV-FD-LFNorm (24h median)	0,61	0,61	0,00
HRV-FD-LFNorm (6h median)	0,61	0,61	0,00
HRV-FD-LFNorm (6h minimum)	0,60	0,60	0,00
HRV-FD-LFNorm (3h rolling mean)	0,60	0,60	0,00
HRV-FD-LFNorm	0,60	0,60	0,00
HRV-FD-LFNorm (12h median)	0,60	0,60	0,00
HRV-FD-LFNorm (24h minimum)	0,60	0,60	0,00
HRV-FD-HFNorm (48h minimum)	0,59	0,59	0,00
HRV-TD-AvgNN (48h median)	0,59	0,59	0,00
HRV-FD-VLF (24h minimum)	0,41	0,59	0,00
HRV-FD-VLF (48h minimum)	0,41	0,59	0,00
HRV-FD-LFNorm (12h minimum)	0,59	0,59	0,00
HRV-FD-HFNorm (48h median)	0,58	0,58	0,00
HRV-FD-VLF (12h minimum)	0,42	0,58	0,00
HRV-FD-HFNorm (24h median)	0,58	0,58	0,00
HRV-TD-AvgNN (24h median)	0,58	0,58	0,00
HRV-TD-AvgNN (3h rolling mean)	0,58	0,58	0,00
HRV-TD-AvgNN (6h median)	0,58	0,58	0,00
HRV-FD-HFNorm (12h minimum)	0,58	0,58	0,00

HRV-TD-AvgNN	0,58	0,58	0,00
HRV-FD-VLF (6h minimum)	0,42	0,58	0,00
HRV-FD-HFNorm (24h minimum)	0,58	0,58	0,00
HRV-FD-HFNorm (12h median)	0,57	0,57	0,00
HRV-FD-HFNorm (6h median)	0,57	0,57	0,00
HRV-FD-HFNorm (6h minimum)	0,57	0,57	0,00
HRV-FD-HFNorm (3h rolling mean)	0,57	0,57	0,00
HRV-FD-HFNorm	0,57	0,57	0,00
HRV-TD-SDNN (48h minimum)	0,43	0,57	0,00
HRV-FD-VLF (6h median)	0,43	0,57	0,00
HRV-FD-VLF (24h median)	0,43	0,57	0,00
HRV-FD-VLF	0,43	0,57	0,00
HRV-FD-VLF (12h median)	0,43	0,57	0,00
HRV-FD-TP (48h minimum)	0,43	0,57	0,00
HRV-TD-AvgNN (12h median)	0,56	0,56	0,00
HRV-FD-VLF (3h rolling mean)	0,44	0,56	0,00
HRV-FD-TP (12h minimum)	0,44	0,56	0,00
HRV-FD-TP (24h minimum)	0,44	0,56	0,00
HRV-FD-VLF (48h median)	0,44	0,56	0,00
HRV-TD-SDNN (24h minimum)	0,44	0,56	0,00
HRV-TD-SDNN (12h minimum)	0,44	0,56	0,00
HRV-TD-TriangularIdx (24h median)	0,44	0,56	0,00
HRV-TD-TriangularIdx (48h minimum)	0,44	0,56	0,00
HRV-FD-TP (6h minimum)	0,44	0,56	0,00
HRV-FD-LF (deviation from expanding mean)	0,44	0,56	0,00
HRV-TD-TriangularIdx (48h median)	0,44	0,56	0,00
HRV-TD-SDNN (6h minimum)	0,44	0,56	0,00
HRV-TD-SDNN (6h median)	0,44	0,56	0,00
HRV-TD-TriangularIdx (24h minimum)	0,44	0,56	0,00
HRV-TD-SDNN (24h median)	0,44	0,56	0,00
HRV-TD-SDNN (12h median)	0,45	0,55	0,00
HRV-FD-TP (12h median)	0,45	0,55	0,00
HRV-TD-SDNN (linear trend (48h))	0,45	0,55	0,00
HRV-TD-TriangularIdx	0,45	0,55	0,00
HRV-TD-TriangularIdx (3h rolling mean)	0,45	0,55	0,00
HRV-FD-TP (6h median)	0,45	0,55	0,00
HRV-FD-TP (24h median)	0,45	0,55	0,00
HRV-TD-SDNN	0,45	0,55	0,00
HRV-TD-TriangularIdx (12h median)	0,45	0,55	0,00
HRV-TD-SDNN (48h median)	0,45	0,55	0,00
HRV-TD-TriangularIdx (6h median)	0,45	0,55	0,00
HRV-FD-TP	0,45	0,55	0,00
HRV-TD-TriangularIdx (12h minimum)	0,45	0,55	0,00
HRV-TD-SDNN (3h rolling mean)	0,45	0,55	0,00

HRV-FD-HF (24h minimum)	0,45	0,55	0,00
HRV-FD-TP (48h median)	0,45	0,55	0,00
HRV-FD-HF (12h minimum)	0,45	0,55	0,00
HRV-FD-TP (3h rolling mean)	0,45	0,55	0,00
HRV-TD-TriangularIdx (6h minimum)	0,45	0,55	0,00
HRV-FD-HF (48h minimum)	0,45	0,55	0,00
HRV-TD-SDNN (deviation from expanding mean)	0,46	0,54	0,00
HRV-TD-ppNN10 (deviation from expanding mean)	0,47	0,53	0,00
HRV-TD-TriangularIdx (deviation from expanding mean)	0,47	0,53	0,00
HRV-FD-HF (12h median)	0,47	0,53	0,00
HRV-FD-LFHFRatio (48h minimum)	0,47	0,53	0,00
HRV-FD-TP (deviation from expanding mean)	0,47	0,53	0,00
HRV-FD-HF	0,47	0,53	0,00
HRV-FD-HF (6h median)	0,47	0,53	0,00
HRV-TD-pNN50 (deviation from expanding mean)	0,47	0,53	0,00
HRV-FD-HF (6h minimum)	0,47	0,53	0,00
HRV-FD-HF (24h median)	0,47	0,53	0,00
HRV-FD-HF (3h rolling mean)	0,48	0,52	0,00
HRV-TD-pNN50 (48h median)	0,52	0,52	0,00
HRV-TD-ppNN10 (24h median)	0,52	0,52	0,00
HRV-FD-LFHFRatio (24h minimum)	0,48	0,52	0,00
HRV-FD-LF (24h minimum)	0,48	0,52	0,00
HRV-TD-AvgNN (linear trend (48h))	0,52	0,52	0,00
HRV-FD-LF (12h minimum)	0,48	0,52	0,00
HRV-FD-HF (48h median)	0,48	0,52	0,00
HRV-TD-SDSD (deviation from expanding mean)	0,48	0,52	0,00
HRV-FD-LFNorm (deviation from expanding mean)	0,48	0,52	0,00
HRV-TD-RMSSD (deviation from expanding mean)	0,48	0,52	0,00
HRV-FD-LF (48h minimum)	0,48	0,52	0,00
HRV-TD-pNN50 (48h minimum)	0,52	0,52	0,00
HRV-FD-LF (12h median)	0,48	0,52	0,00
HRV-FD-VLF (deviation from expanding mean)	0,48	0,52	0,00
HRV-TD-ppNN10 (48h median)	0,52	0,52	0,00
HRV-FD-LF	0,48	0,52	0,00
HRV-TD-pNN50 (24h median)	0,52	0,52	0,00
HRV-TD-ppNN10 (3h rolling mean)	0,52	0,52	0,00
HRV-FD-LFHFRatio (48h median)	0,49	0,51	0,00
HRV-FD-LF (24h median)	0,49	0,51	0,00
HRV-TD-SDSD (48h median)	0,51	0,51	0,00
HRV-TD-RMSSD (48h median)	0,51	0,51	0,00
HRV-FD-LFHFRatio (12h minimum)	0,49	0,51	0,00
HRV-FD-HFNorm (deviation from expanding mean)	0,51	0,51	0,00

HRV-FD-LF (6h median)	0,49	0,51	0,00
HRV-FD-LF (3h rolling mean)	0,49	0,51	0,00
HRV-FD-LFHFRatio (6h median)	0,49	0,51	0,00
HRV-TD-ppNN10 (6h median)	0,51	0,51	0,00
HRV-FD-LF (6h minimum)	0,49	0,51	0,00
HRV-TD-RMSSD (linear trend (48h))	0,49	0,51	0,00
HRV-FD-HF (deviation from expanding mean)	0,49	0,51	0,00
HRV-TD-pNN50 (3h rolling mean)	0,51	0,51	0,00
HRV-FD-LFHFRatio (6h minimum)	0,49	0,51	0,00
HRV-TD-ppNN10 (12h median)	0,51	0,51	0,00
HRV-TD-ppNN10	0,51	0,51	0,00
HRV-TD-RMSSD (3h rolling mean)	0,51	0,51	0,00
HRV-TD-SDSD (3h rolling mean)	0,51	0,51	0,00
HRV-TD-SDSD (24h minimum)	0,49	0,51	0,00
HRV-TD-RMSSD (24h minimum)	0,49	0,51	0,00
HRV-TD-pNN50	0,51	0,51	0,00
HRV-TD-SDSD (12h minimum)	0,49	0,51	0,00
HRV-TD-RMSSD (12h minimum)	0,49	0,51	0,00
HRV-FD-LFHFRatio	0,49	0,51	0,00
HRV-TD-SDSD (24h median)	0,51	0,51	0,00
HRV-TD-RMSSD (24h median)	0,51	0,51	0,00
HRV-TD-pNN50 (6h median)	0,51	0,51	0,00
HRV-FD-LFHFRatio (3h rolling mean)	0,49	0,51	0,01
HRV-TD-AvgNN (deviation from expanding mean)	0,51	0,51	0,01
HRV-TD-pNN50 (12h median)	0,51	0,51	0,03
HRV-TD-RMSSD	0,51	0,51	0,03
HRV-TD-SDSD	0,51	0,51	0,03
HRV-FD-LFHFRatio (12h median)	0,49	0,51	0,04
HRV-FD-LFHFRatio (24h median)	0,49	0,51	0,05
HRV-TD-pNN50 (6h minimum)	0,49	0,51	0,02
HRV-TD-pNN50 (12h minimum)	0,51	0,51	0,01
HRV-FD-LFHFRatio (deviation from expanding mean)	0,49	0,51	0,09
HRV-TD-RMSSD (6h median)	0,50	0,50	0,12
HRV-TD-SDSD (6h median)	0,50	0,50	0,13
HRV-TD-SDSD (48h minimum)	0,50	0,50	0,14
HRV-TD-RMSSD (48h minimum)	0,50	0,50	0,14
HRV-TD-AvgNN (hourly change)	0,50	0,50	0,14
HRV-TD-SDSD (6h minimum)	0,50	0,50	0,19
HRV-TD-RMSSD (6h minimum)	0,50	0,50	0,20
HRV-FD-LF (48h median)	0,50	0,50	0,20
HRV-TD-RMSSD/AvgNN ratio	0,50	0,50	0,36
HRV-TD-pNN50 (24h minimum)	0,50	0,50	0,18
HRV-TD-TriangularIdx (hourly change)	0,50	0,50	0,49
HRV-TD-SDSD (hourly change)	0,50	0,50	0,50

HRV-FD-VLF (hourly change)	0,50	0,50	0,51
HRV-TD-RMSSD (hourly change)	0,50	0,50	0,52
HRV-FD-LFHFRatio (hourly change)	0,50	0,50	0,54
HRV-TD-ppNN10 (hourly change)	0,50	0,50	0,45
HRV-FD-TP (hourly change)	0,50	0,50	0,70
HRV-TD-SDSD (12h median)	0,50	0,50	0,76
HRV-TD-RMSSD (12h median)	0,50	0,50	0,82
HRV-FD-LFNorm (hourly change)	0,50	0,50	0,82
HRV-TD-ppNN10 (6h minimum)	0,50	0,50	0,02
HRV-FD-HFNorm (hourly change)	0,50	0,50	0,87
HRV-TD-SDNN (hourly change)	0,50	0,50	0,89
HRV-TD-ppNN10 (12h minimum)	0,50	0,50	0,01
HRV-FD-HF (hourly change)	0,50	0,50	0,91
HRV-TD-ppNN10 (48h minimum)	0,50	0,50	0,38
HRV-TD-pNN50 (hourly change)	0,50	0,50	0,98
HRV-FD-LF (hourly change)	0,50	0,50	0,99
HRV-TD-ppNN10 (24h minimum)	0,50	0,50	0,98

Table S3: Baseline characteristics of included versus excluded patients. Comparison of the 101 patients included in the analysis (continuous ECG of sufficient quality and duration for HRV computation) with the 238 patients excluded for insufficient ECG availability, across all baseline characteristics reported in the main cohort table. Values are median (IQR) for continuous and ordinal variables and n (%) for binary variables. Percentages for binary variables are computed within each column. P values are from the Mann–Whitney U test (continuous/ordinal) or Fisher's exact test (binary), testing the null hypothesis of no between-group difference. SMD denotes the absolute standardized mean difference, a sample-size–independent measure of between-group imbalance. Demographics and comorbidities were comparable between groups, whereas the included cohort showed higher admission neurological severity (Hunt & Hess, WFNS, modified Fisher, extracerebral GCS). Critically, the prevalence of the prediction target (DCI) did not differ between groups (34.7% vs 39.1%, p = 0.47), preserving the outcome case mix. GCS, Glasgow Coma Scale; WFNS, World Federation of Neurosurgical Societies grade; mFS, modified Fisher Scale; BNI, Barrow Neurological Institute grade; DCI, delayed cerebral ischaemia; IQR, interquartile range; SMD, standardized mean difference.

**Characteristic**	**All (n=339)**	**Included (n=101)**	**Excluded (n=238)**	**p value**	**SMD**
"Age in years, median (IQR)"	58.0 (49.0-68.0)	59.0 (50.0-66.0)	57.0 (49.0-69.0)	0.924	0.030
"Female sex, n (%)"	218 (64.9)	69 (68.3)	149 (63.4)	0.455	0.104
"Hypertension, n (%)"	126 (37.2)	40 (39.6)	86 (36.1)	0.542	0.072
"Cardiovascular disease, n (%)"	60 (17.8)	18 (18.0)	42 (17.6)	1.000	0.009
"Diabetes, n (%)"	20 (5.9)	8 (7.9)	12 (5.0)	0.319	0.117

"GCS (extracerebral), median (IQR)"	13.0 (8.0-15.0)	12.0 (6.0-14.0)	14.0 (9.0-15.0)	0.002	0.309
"Hunt & Hess, median (IQR)"	3.0 (2.0-4.0)	3.0 (2.0-4.0)	2.0 (2.0-3.8)	<0.001	0.430
"Hunt & Hess 4-5, n (%)"	97 (28.6)	37 (36.6)	60 (25.2)	0.036	0.249
"WFNS, median (IQR)"	2.0 (1.0-4.0)	4.0 (2.0-5.0)	2.0 (1.0-4.0)	0.002	0.363
"WFNS 4-5, n (%)"	143 (42.2)	52 (51.5)	91 (38.2)	0.030	0.269
"mFS, median (IQR)"	4.0 (3.0-4.0)	4.0 (3.0-4.0)	4.0 (3.0-4.0)	<0.001	0.431
"mFS 3-4, n (%)"	308 (90.9)	98 (97.0)	210 (88.2)	0.012	0.342
"BNI, median (IQR)"	3.0 (3.0-4.0)	3.0 (3.0-4.0)	3.0 (3.0-4.0)	0.530	0.083
"BNI 4-5, n (%)"	157 (46.3)	50 (49.5)	107 (45.0)	0.476	0.091
"Hydrocephalus at admission, n (%)"	169 (49.9)	59 (58.4)	110 (46.2)	0.044	0.246
"DCI, n (%)"	128 (37.8)	35 (34.7)	93 (39.1)	0.465	0.092

Figure S1: Impurity-based (mean decrease in impurity) feature importance from the COMBINED model, which integrates static admission variables (Static), laboratory/blood-gas measurements (LAB/BGA), and heart-rate variability (HRV) features. Left: the 20 highest-ranked individual features, with bars coloured by modality (grey = static, green = LAB/BGA, orange = HRV). The x-axis is the mean feature importance. The single strongest predictor is a blood-gas variable (bga_Cl^{-}), but HRV features occupy the majority of the top-20 (11 of 20), including low-/high-frequency power (hrv_fd_lf_norm, hrv_fd_hf_norm) and time-domain measures and their 48 h trends (hrv_dt_avgnn, hrv_dt_rmssd, hrv_dt_sdnn). Right: importance aggregated (summed) by modality, showing that HRV accounts for the largest share of total model importance (HRV 55.8%, LAB/BGA 37.9%, static 6.2%).

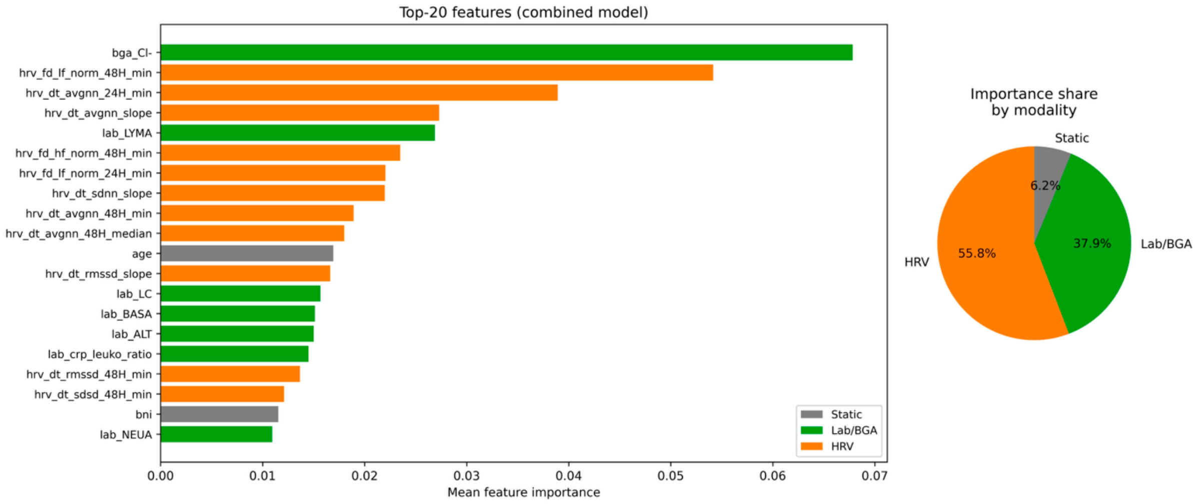

Figure S2: Robustness of modality-level feature importance in the combined model. Feature importance attributed to each input modality (static admission variables as <u>Static</u>, laboratory/blood-gas measurements as <u>LAB/BGA</u>, and heart-rate variability as <u>HRV</u>) assessed with two complementary methods to address the known instability and feature-count bias of impurity-based importance in correlated, high-dimensional feature sets. (A) Impurity-based (mean decrease in impurity, MDI) importance share per modality. Bars show the mean share across the five outer cross-validation folds and dots show the five individual fold values. The dashed line marks 50%. HRV accounted for a median 59% of total importance (95% CI [51.7, 70.3]), exceeding 50% and ranking first in all 5/5 folds. (B) Grouped permutation importance, quantified as the drop in patient-level AUC when an entire modality block is jointly permuted on held-out data. Error bars are 95% confidence intervals. Permuting the HRV block reduced AUC by 0.111 ([0.0024, 0.2127]) versus 0.056 for LAB/BGA and ≈0 for Static, i.e. HRV accounted for ~66% of the total permutation importance. Because the whole block is permuted jointly, this estimate is robust to within-modality correlation and is not inflated by the larger number of HRV features (173). The two methods agree that HRV is the dominant modality, and this ranking is stable across folds. Given the limited number of DCI events (n = 34), per-fold permutation CIs are wide, so the consistency of the ranking should be emphasised over the precise magnitude.

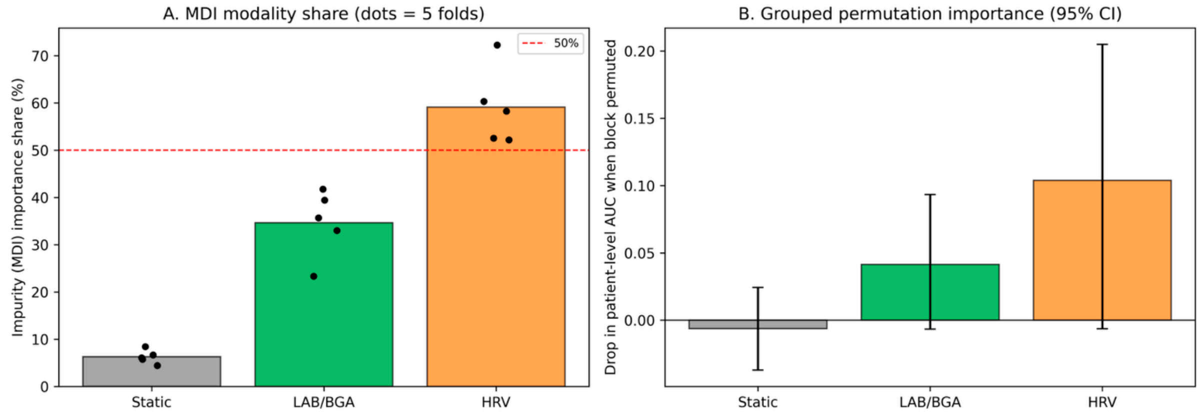

Figure S3. Time-resolved added value of HRV over the LAB/BGA model. Discrimination is evaluated within five event-balanced intervals of days since symptom onset. The x-axis shows equal-event quintiles with interval boundaries at 2.0/4.7/6.1/7.5/9.0/13.1 days. (A) Patient-level ROC AUC of the LAB/BGA model versus the COMBINED model (LAB/BGA + HRV + static admission variables) within each interval. The dashed line marks chance (0.5). LAB/BGA discriminates well early (AUC 0.718 at 2.0–4.7 days) and declines thereafter, whereas the COMBINED model is weakest early (0.534) but rises to overtake LAB/BGA in the latest interval (0.681 vs 0.568 at 9.0–13.1 days). (B) Incremental discrimination per interval, expressed as ΔAUC = COMBINED − LAB/BGA (bars; green where adding HRV improves discrimination, red where it degrades it). Adding HRV reduced discrimination in the two earliest intervals (ΔAUC −0.184, the only interval reaching significance, and −0.075) and improved it in the later intervals (+0.096, +0.006, +0.113). Overlaid markers show the same COMBINED − LAB/BGA increment under three alternative model specifications (best-per-feature-set, count-matched random forest, count-matched HistGB). The late-interval benefit was positive under all three (+0.124, +0.148, +0.103), confirming the early-harm/late-benefit pattern is not specific to one architecture. The added value of HRV over routine laboratory/blood-gas features is therefore concentrated in the late clinical phase (days 9–13.1). Given the limited number of DCI events, per-interval confidence intervals are wide (late interval 95% CI [−0.021, +0.269]). The consistency of the late benefit across architectures should be emphasised over its precise magnitude.

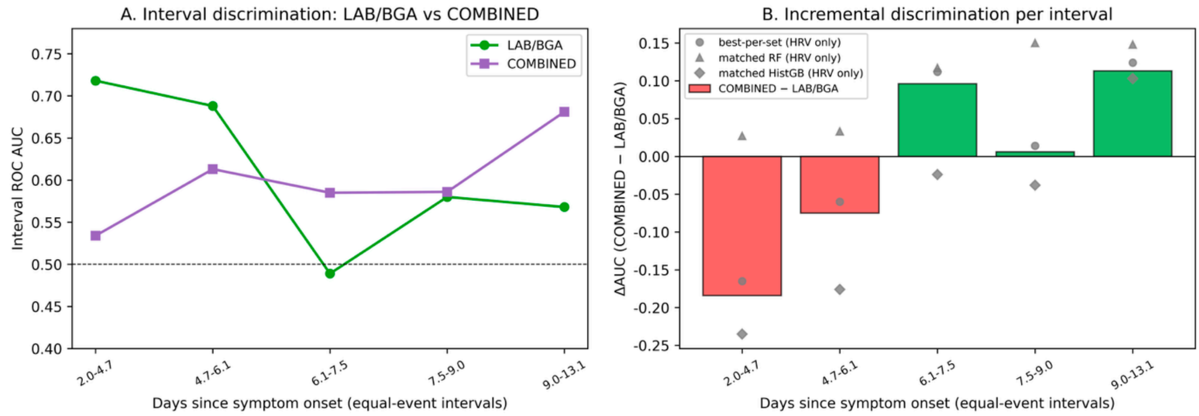

Supplement: Supplementary file 1 [file bioengineering-13-00857-s001.zip › bioengineering-4446766-supplementary.pdf]
